# Supplementary material for: 4D nanoimaging of early age cement hydration
Source: Nat Commun. 2023 May 8;14:2652. doi: 10.1038/s41467-023-38380-1 (PMC10167225; doi:10.1038/s41467-023-38380-1)
Supplement: Supplementary file 1 — Supplementary Information [file 41467_2023_38380_MOESM1_ESM.pdf]

Supplementary Information

4D nanoimaging of early age cement hydration

Shiva Shirani[1], Ana Cuesta[1], Alejandro Morales-Cantero[1], Isabel Santacruz[1], Ana Diaz[2], Pavel Trtik[3], Mirko Holler[2], Alexander Rack[4], Bratislav Lukic[4], Emmanuel Brun[5], Inés R. Salcedo[6] and Miguel A. G. Aranda[1]*

[1]Departamento de Química Inorgánica, Cristalografía y Mineralogía, Universidad de Málaga, 29071 Málaga, Spain.
[2]Laboratory for Macromolecules and Bioimaging, Paul Scherrer Institut, 5232 Villigen PSI, Switzerland.
[3]Laboratory for Neutron Scattering and Imaging, Paul Scherrer Institut, 5232 Villigen PSI, Switzerland.
[4]ESRF-The European Synchrotron, 71 Rue des Martyrs, 38000 Grenoble, France.
[5]Université Grenoble Alpes, Inserm UA7 STROBE, 38000 Grenoble, France.
[6]Servicios Centrales de Apoyo a la Investigación, Universidad de Málaga, 29071 Málaga, Spain.

*Corresponding author: g_aranda@uma.es

In the format provided by the authors and unedited

Table of contents:

Supplementary Contents		Description	Page
Supplementary Methods	Methods	Comprehensive methods description	3-6
All Data	Open access data	Tomography and laboratory raw data	7
Supplementary Tables	Table S1	Chemical analysis by X-ray fluorescence	8
	Table S2	Rietveld quantitative phase analysis of the Portland cements	8
	Table S3	Textural details for the two cements	8
	Table S4	Selected cumulative heat release data from isothermal calorimetry	8
	Table S5	Rietveld quantitative phase analysis for the hydrating paste	9
	Table S6	Mean β values converted to μ, and the resulting w/c ratios	9
	Table S7	Mass and electron densities values for selected components	10
	Table S8	Component segmentation and average electron densities from PXCT	11
	Table S9	Volume percentages for the hydrating pastes by the used techniques	12
Supplementary Figures	Figure S1	Laboratory Rietveld plots for the anhydrous cements	13
	Figure S2	Lab-μCT selected orthoslice and grey-value profile	14
	Figure S3	Syn-μCT selected orthoslice and grey-value profile	14
	Figure S4	PXCT selected orthoslice and grey-value profile	15
	Figure S5	Lab-μCT Fourier Shell Correlation plots	16
	Figure S6	Syn-μCT Fourier Shell Correlation plots	17
	Figure S7	PXCT Fourier Shell Correlation plots	18
	Figure S8	PXCT electron density and absorption orthoslices	19
	Figure S9	Bivariate histograms of electron densities and absorption indexes	20
	Figure S10	VOI histogram of the electron densities for PXCT	20
	Figure S11	Second etch-pit evolution picture	21
	Figure S12	PXCT vertical views showing the paste evolution	22
	Figure S13	Etch-pit growth rates variability	23
	Figure S14	2D view of PXCT and electron density profile, first example	24
	Figure S15	Alite dissolution and C-S-H gel densification for the first example	24
	Figure S16	2D view of PXCT and electron density profile, second example	25
	Figure S17	Alite dissolution and C-S-H gel densification for the second example	25
	Figure S18	2D view of PXCT and electron density profile, third example	26
	Figure S19	2D view of PXCT and electron density profile, fourth example	26
	Figure S20	PXCT vertical views showing the water/air porosity evolution	27
	Figure S21	Machine Learning training flow chart	28
	Figure S22	C-S-H shell segmentation flow chart	29
	Figure S23	Comparison of the C-S-H shell raw data and segmentation output	29
	Figure S24	PXCT orthoslices showing water/air porosity evolution, second example	30
	Figure S25	PXCT orthoslices showing water/air porosity evolution, third example	30
	Figure S26	PXCT orthoslices showing selected features of the paste evolution	31
	Figure S27	PXCT vertical views for a fast dissolving particle	31
	Figure S28	Capillary water porosity evolution	32
Supplementary Movies	Movie- 1	Summary of 4D nanoimaging of cement hydration	33
	Movie- 2	C-S-H shell characterization at 19 hours	33
Article Cover Image	Cover	X-ray nanoimaging of a hydrating cement paste at early ages	33
Supplementary References	References	Additional references	34

- **Supplementary Methods**

Synchrotron X-ray computed microtomography experiment (Syn-μCT).

Microtomographic scans were acquired at the 150 m-long beamline ID19 of the European Synchrotron (ESRF) in Grenoble, France. A so-called single-harmonic undulator (type: u17.6, gap 16.5 mm) was chosen as a source due to its excellent photon flux density at a narrow bandwidth around approximately 19 keV photon energy. The u17.6 allows beamline ID19 to be operated only with the two mandatory windows (0.8 mm diamond in the front-end and 0.5 mm Beryllium in the experimental hutch) and an 0.7 mm-thick Aluminium attenuator and hence, guaranties a homogeneous wave front: which is suited for high-sensitivity measurements by means of inline propagation-based phase contrast. The propagation distance between sample and detector was set to 15 mm. The indirect high-resolution detector consisted of a so-called revolver-microscope by the French company OptiquePeter (Lentilly, France)[1], the system lens-couples an 8.7 μm-thin LSO:Tb (Tb-doped Lu_2SiO_5) single-crystal scintillator with a 10× Olympus microscope (0.3NA) to a sCMOS-based camera (type: pco.edge, PCO AG, Germany)[2]. The effective pixel size of the detector assembly is approximately 0.6 μm. 6000 projection angles were acquired over a 360 degree tomographic scan with an exposure time of 0.05 s, i.e. 5 minutes scan, at ~21.5 °C. During this experiment, the ESRF operated in so-called 4bunch timing mode with a reduced ring current of maximum 20 mA. The estimated flux density at the sample position was 4.2×10^{11} photons$\cdot s^{-1}\cdot mm^{-2}$. Phase retrieval of the projections was performed using the Paganin algorithm[3], considering the ratio of the refractive and absorption index δ/β equal to 70. In order to retrieve the microstructural content introduced by the inherent smoothing characteristics of the phase retrieval method, a Gaussian unsharp mask was applied. The voxel size with the employed configuration, to fully image a capillary of 0.7 mm of diameter, was 0.65 μm.

The tomographic reconstructions were performed using the open-source tomography software available at the ESRF, relying on the sub-packages NXtomoMill and NABU[4]. Given its straightforward Graphics Processing Unit (GPU)-based implementation, the full volume reconstructions were performed on the Power9 cluster using the gold-standard filtered back projection (FBP) algorithm. The projections are first corrected for beam profile illumination (flat field), dark current noise of the detector (dark field) and filtered for any potential pixel outliers arising from stray photons. The reconstructed volume, consisting of 2490×2490×1950 pixels, is cast to 16 bit format considering the 10-90% of the volume histogram and cropped to the region of interest. In the case of the reconstructions used for the Fourier Shell Correlation (FSC) analysis,[5] the original reconstruction is split into two sub-sampled reconstructions considering the number of the reconstructed either even or odd projections.

Near-field ptychographic X-ray computed tomography (PXCT).

The measurements were carried out with a high-stability instrument designed for high-resolution PXCT working in air and at room temperature[6,7], using a photon energy of 8.93 keV. The coherent illumination was defined with a Fresnel zone plate (FZP) of 120 μm diameter and 60 nm outer-most-zone width, which at this energy had a focal distance of 51.9 mm. The FZP had locally displaced zones, specifically designed to produce an optimal illumination for ptychograph[8]. The flux of the X-ray beam was 1.7×10^{8} photons$\cdot s^{-1}$ at the sample position. The sample was placed at 13 mm downstream the focus, where the illumination had a size of about 30 μm. Ptychographic scans were recorded following the positions of a Fermat spiral[9] with an average step size of 6 or 7 μm and a field of view of 186 μm × 30 μm (horizontal × vertical). The field of view must be larger than the size of the capillary to include an air region at both sides of the sample, which is needed for successful tomographic reconstructions and for quantitative contrast. At each scanning position, magnified images of the sample were recorded with an in-vacuum Eiger 1.5M detector[10] with a pixel size of 75 μm placed at 5.237 m downstream the sample, with an acquisition time of 0.1 s. A scan speed of ~5 Hz was achieved thanks to a combined motion of the FZP and the sample, while achieving an effective static illumination on the sample during acquisition[11]. Near-field ptychographic scans were repeated at 420 rotation angles of the sample in equal intervals from 0 to 180 deg. We recorded a total of 3 tomograms at different times from the start of the cement hydration at ~25 °C, the temperature of the

experimental hutch. The first tomogram was recorded with an average scanning step size of 6 μm, it started at 17 h and finished at 20h and 55 minutes, after water mixing, i.e. 3h 55 min of total acquisition time. This scan is hereafter labelled 19 h dataset. The other two tomograms were recorded with a step size of 7 μm lasting 3h 6 min. The scans labelled 47 and 93 h started at 46 and 92 h (after water mixing), respectively. The scan times include the dead time during motion of stages in between acquisitions. The dose absorbed by the specimen during data acquisition was estimated to be 0.7 and 0.5 MGy for the tomograms with 6 and 7 μm of step size, respectively.

Near-field ptychographic reconstructions were performed for each projection using the Ptycho Shelves package[12] developed by the Coherent X-ray Scattering group at PSI, using 5000 iterations of a difference map algorithm[13] adapted for near-field geometry. The pixel size of the images, determined by geometric magnification, is 186.64 nm and we estimate by Fourier ring correlation[5] that the 2D resolution of each reconstructed image is about 200 nm. For each tomographic dataset, projections were aligned with sub-pixel accuracy and processed for phase tomographic reconstruction from phase projections as previously reported[14,15]. The 3D spatial resolution was estimated by FSC[5]. The resolution obtained, see subsection dedicated to the spatial resolution, was limited by the number of projections, which was chosen to have reasonable scan times.

PXCT provides 3D maps of the electron density of the specimen with quantitative contrast, the sensitivity being about 0.02 $e^{-}Å^{-3}$.[16] For attaining quantitative electron densities, the entire specimen must be included in the field of view, containing some empty space around it, which was the case in our measurements. Therefore, it is possible to easily distinguish air and water regions in the specimen, which have electron densities of 0.00 and 0.33 $e^{-}Å^{-3}$, respectively. Obviously, neutron imaging is the standard technique to disentangle water from air porosities.[17] A key advantage of neutron imaging is its ability to can scan large volumes. However, it must also be noted that at inferior spatial resolution compared to PXCT.

Tomographic data analysis.

Initially, the re-alignment of the data, when needed must be detailed.

For the PXCT, the capillary position was very accurate, as the capillary/holder system was mounted from the tray storage to the sample stage by the flOMNY gripper (robot). Hence, the angular orientation of the sample was maintained. The field of view of the sample was aligned carefully based on features visible in the 2D projections. The scanned regions with time were consistent within a few voxels and therefore no alignment between different acquisitions was required.

For the Syn-μCT, a mark was drawn on the sample holder and sample stage for the incident beam to minimise the initial incidence angular position variability. Before each scan, a projection was acquired as a reference for the next one in order to scan the same region. A minor manual registration was required, mostly rotations around x- and y-axes.

For the Lab-μCT, manual registration was required to align the different acquisitions. The process is described next. The capillary was considered as a cylinder and we manually made the cylinders vertical and centred in the reconstructed volume. The remaining rotation around the z-axis was visually done by superimposing distinguishable landmarks in the corresponding images.

The segmentation was done on a Volume of Interest (VOI) corresponding to the inner part of the capillaries for each imaged sample. The total volume of these VOIs varies depending on the sample sizes, amounting to ~1×10^5 $μm^3$ for each PXCT dataset and ~1×10^8 $μm^3$ for Syn-μCT and Lab-μCT samples. A supervised Machine-Learning (ML) image analysis approach was used to segment the different components of the scanned samples, using the IPSDK Explorer software (version 3.2.0.0 for Windows™, Reactiv'IP, Grenoble, France). This software allows us to manually label voxels on a selected training dataset (approximately 31 voxels for each component on average for PXCT, 20 voxels on average for Syn-μCT and Lab-μCT) and to rapidly obtain test results to determine if the labelling is sufficient or if it requires more information/re-training. The initial classification was based on the electron densities with a variation of ~5% of the measured values, from selected volumes, which are given in Table S7. These test results are obtained after

a first learning step using a random forest method. It is also possible for the user to keep or remove features used in the random forest decision trees based on their relevance. This method permitted to segment the components with comparable grey values and/or electron densities, overlaid ML models on raw datasets are shown in Fig. 7.

On the one hand, the good contrast and the high spatial resolution in PXCT allowed to classify the components into seven categories. They are given next from higher to lower electron densities: i) C_4AF (yellow) with highest values; ii) $C_3S/C_2S/C_3A$ (dark brown) which are the clinker particles; iii) calcite (pink); iv) portlandite (green); v) the rest of the hydrated phases with lower electron densities were labelled as 'Low-Density Hydrates' (light brown), i.e. C-S-H gel, iron-silicon-hydrogarnet, hemicarbonate and ettringite; vi) water porosity (blue); and vii) air porosity (black). On the other hand, due to the contrast and spatial resolution limitations in the two other modalities, Syn-μCT and Lab-μCT, the components were classified into four categories. The classification from higher to lower grey-values was: i) clinker particles (dark brown), i.e. $C_4AF/C_3S/C_2S/C_3A$; ii) a component labelled 'High-Density Hydrates' (green), being mainly portlandite and calcite; iii) another component labelled 'Low-Density Hydrates' (light brown), being mainly C-S-H gel, iron-silicon-hydrogarnet, hemicarbonate and ettringite; and iv) porosity (black) which contain both water and air. It is noted that Syn-μCT and Lab-μCT microtomographies do not allow to distinguish water from air porosities due to the similarities in their X-ray attenuation values.

This ML approach also permits to mitigate the influence of partial volume effects in-between labelled components for accurate quantitative analysis of PXCT, i.e. mean electron density. Selected results after the PXCT segmentation procedure are summarised SI. Movie 1. In addition, after grains were segmented using the ML approach described above, the C-S-H gel shell thickness was computed on PXCT imaged sample at 19 h, see Fig. 6 and SI. Movie 2. The wall thickness script computes the object thickness. For a given pixel, the thickness is the radius of the largest circle centred on this pixel entirely included in the object. The steps of the data analysis process are shown in flowcharts, see Fig. S20 and S21. A further post-segmentation data analysis calculation was carried out in order to show the particle size distribution evolution with hydration time. The anhydrous cement particles, at the three hydration times, were classified by computing their mean Feret diameters. Fig. 8b displays the volume percentage of the segmented grains (and their cumulative volumes) as function of the particle sizes that can be compared with the initial characterization by laser diffraction, see Fig. 1a. 3D rendering visualization was done using Dragonfly software (version 2022.1 for Windows™, Object Research Systems (ORS) Inc., Montreal, Canada).

Spatial resolution analysis.

The spatial resolution was characterised by two approaches as recently reported[18]. On the one hand, it can be determined from the grey-value changes in line profiles through the edge sharpness of the interfaces. A point spread function (PSF) used to determine the spatial resolution of the images as ISO/TS 24597 defines the Gaussian radius of the PSF as the resolution, which equals to a change between 25 %–75 % grey value along the studied interfaces.[19] Here, a common interface present in the three imaging modalities has been selected for the line profiles: the glass capillary wall – air (i.e. exterior of the capillaries). We have measured 25 interfaces in every tomogram, which allowed us to determine the average spatial resolution and its associated standard deviation. Moreover, as examples, Figures S2-S4 display line profiles of sharp interfaces between high (i.e. alite) and low density (i.e. porosity) components within the capillaries. The spatial resolutions, as determined by this approach, were 250(25) nm, 264(25) nm, 272(34) nm, 748(19) nm and 2.21(17) μm, for PXCT-19h, PXCT-47h, PXCT-93h, Syn-μCT and Lab-μCT datasets, respectively.

On the other hand, FSC plots[5] have been also computed. The traces are displayed in Figures S5-S7 giving spatial resolution values of 430 nm, 470 nm, 500 nm, 650 nm and 1.9 μm, for PXCT-19h, PXCT-47h, PXCT-93h, Syn-μCT and Lab-μCT datasets, respectively. Moreover, the FSC trace for PXCT-19h shows a smooth decrease in the 0.0-0.2 spatial frequency range, which is likely due to the hydration of cement during the 4-hour measurement. As expected, this behaviour is not shown at later ages.

It should be noted that the agreement between the spatial resolution results between the edge sharpness approach and FSC method is satisfactory for Syn-μCT (750 vs. 650 nm) and Lab-μCT (2.2 vs.1.9 μm) datasets.

However, the agreement between these two approaches is not good for PXCT (for instance, 250 vs 430 nm at 19 h). The poorer resolution estimated by FSC can be explained because the angular sampling is very tight, i.e. 420 projections, so the two employed subtomograms in the FSC, each of 210 projections, were significantly undersampled compared to the number of voxels across the diameter of the sample. This means that the correlation between two such undersampled tomograms can give a low estimation of the spatial resolution. This feature is not observed for Syn-μCT and Lab-μCT as the total number of projections were 6000 and 1637, respectively. In other words, the subtomograms with half the number of projections were not undersampled for these two imaging modalities.

Etch-pit growth rate evaluation.

The estimation of the etch-pit growth rate was based on the analysis of 27 etch-pits from 5 different large alite grains. It is noted that the etch-pits have irregular 3D shapes and therefore, for its spatial dissolution rate estimation, some simplifications were undertaken. Moreover, the spatial resolution of this PXCT work, ~250 nm, is limited for accurate analyses. Therefore, we consider this approach as an estimation. Firstly, etch-pits were visually selected from grains with sizes larger than 10 μm. Secondly, their overall shapes were compared in two hydrating steps. Then, two envelopes from pixels with at least 90% of the electron density of alite were developed. The estimated/calculated distance (in pixels) was computed between these edges for the deepest perpendicular length. These values were converted to dissolution rate by taking the ratio respect to the time between measurements. The result for the analysis between 19 and 47 h datasets gave 6.1 pixels of average distance which is equivalent to 41(29) nm/h. There was large variability in the rates, the fastest being 110 nm/h and the slowest being 10 nm/h. From this investigation, it is not possible to know if this large variability comes from the heterogeneity in the defects within these regions, or if other variables like the spatial resolution of this work and the local water-to-cement ratio variations are also playing important roles. More imaging studies are necessary to establish this. The very same 27 etch-pits were also analysed between 47 and 93 h datasets. In this case, the etch-pit growth rate was slower 7 nm/h, showing that the water diffusion is already limiting hydration at four days.

Water/cement ratio estimation of the scanned sample by PXCT

The w/c ratios of the scanned capillaries in a selected region can be calculated at the different ages according to the procedure previously reported[20]. The final β-mean values obtained by PXCT were used after converting to μ values, see Table S6. Then, using the mineralogical compositions of the anhydrous cement (given in the Supporting information, Table S2) the μ value is estimated, taking into account the μ value of free water, 22.2 cm^{-1}. For instance, for the 19 h sample, it can be estimated that the paste was composed of 69.9 wt% PC and 30.1 wt% water to account for the overall μ of the paste. This calculation yielded a w/c ratio of 0.39, see Table S6.

Chemical reactions

I. The chemical reactions used for the FW calculation, see Table S5, are: (1) the consumption of water by the hydration of C_4AF, with C_3S which is the source of silicates, to give amorphous iron siliceous hydrogarnet (Fe-Si-Hg) and crystalline portlandite; (2) the hydration of C_3S to yield amorphous C-S-H gel and crystalline portlandite; (3) the hydration of C_3A, consuming a calcium sulfate source, to yield ettringite if there are enough sulfates available (which is the case here); and (4) the possible carbonation of portlandite gives crystalline (and amorphous) calcium carbonate(s) and it releases free (capillary) water.

II. In the absence of belite hydration, the chemical reaction contributing to C-S-H gel formation, see Table S5, is just (2). It is underlined that a small fraction of the consumed alite did not result in C-S-H gel but in the formation of iron siliceous hydrogarnet from the ferrite hydration (reaction 1).

It is noted here that for the calculations presented in Table S5, the amount of C_3S which is needed for the silicate groups in iron-silicon-hydrogarnet, is calculated first from the degree of hydration of C_4AF (applying reaction #1). Then, the portlandite and C-S-H gel contents are determined from the reaction of C_3S after subtracting the number obtained in the process described just above.

$Ca_4Al_2Fe_2O_{10}$ + $1.68Ca_3SiO_5$ + $11.68H_2O$	→ $2Ca_3AlFe(SiO_4)_{0.84}(OH)_{8.64}$ (amorph.) + $3.04Ca(OH)_2$	(1)
Ca_3SiO_5 + $5.2H_2O$	→ $1.2Ca(OH)_2$ + $(CaO)_{1.8}SiO_2(H_2O)_{4.0}$	(2)
$Ca_3Al_2O_6$ + $3CaSO_4{\cdot}2H_2O$ + $26H_2O$	→ $Ca_6Al_2(SO_4)_3(OH)_{12}{\cdot}26H_2O$	(3)
$Ca(OH)_2$ + CO_2	→ $CaCO_3$ + H_2O	(4)

Open access raw data availability and description

The following raw data has been openly deposited on Zenodo and can be acceded at: https://doi.org/10.5281/zenodo.7030107

1. Tomographic reconstructed raw data of all the X-ray imaging modalities (twelve tomograms) in 16 bit and .tif format. The size of the files is also given in the following table.

Folder Name	Sub-folders Label	File Size*	Additional Information
Phase-PXCT	1_PXCT-19h<br>2_PXCT-47h<br>3_PXCT-93h	736.9MB	4D Synchrotron Ptychographic X-ray Computed Tomography Cement Hydration (Delta Dataset)
Syn-microCT	4_Syn-microCT-19h<br>5_Syn-microCT-47h<br>6_Syn-microCT-93h	52.8GB	4D Synchrotron Phase-contrast Microtomography Cement Hydration
Lab-microCT	7_Lab-microCT-19h<br>8_Lab-microCT-47h<br>9_Lab-microCT-93h	7.5GB	4D Laboratory Attenuation-contrast Microtomography Cement Hydration
Absorption-PXCT	10_abs-PXCT-19h<br>11_abs-PXCT-47h<br>12_abs-PXCT-93h	722.9MB	Absorption Dataset for 4D Synchrotron Ptychographic X-ray Computed Tomography Cement Hydration (Beta Dataset)

Net total size of all datasets: **82.8GB**

**These are zipped files. The original file size for every single 16 bit Syn-microCT dataset is 22.8GB, for each Lab-microCT dataset is 3.0GB and for each PXCT dataset is 500MB.*

2. Laboratory raw data

2.1. Particle size distribution (PSD)

"PSD" labelled folder contains two files in .mmes format.

2.2. Isothermal calorimetry

"Calorimetry" labelled folder contains six files in .xlsx excel format.

2.3) Laboratory X-ray powder diffraction (LXRPD)

"LXRPD" labelled folder contains five files in .ASC text format.

• Supplementary Tables

Table S1. Chemical (elemental) analysis (by X-ray fluorescence) of the two employed Portland cements in this investigation. All data expressed in weight percentages of the corresponding oxides.

	CaO	SiO_2	SO_3	Al_2O_3	Fe_2O_3	MgO	K_2O	Na_2O	Others	LoI
**PC-52.5**	62.2	20.4	3.6	4.9	3.2	1.5	1.1	0.3	0.5	2.3
**PC-42.5**	62.9	19.7	3.4	5.0	3.4	1.5	1.1	0.3	0.3	2.7

Table S2. Rietveld quantitative phase analysis of the employed anhydrous Portland cements, in wt%.

	C_3S	β-C_2S	C_4AF	o-C_3A	$C\bar{S}H_2$	$C\bar{S}H_{0.5}$	Cc	Q	$K\bar{S}$	CaO
**PC-52.5**	61.0	11.8	11.5	8.2	1.9	2.0	2.8	0.8	-	-
**PC-42.5**	61.6	11.1	10.8	7.8	0.9	2.1	3.7	0.6	1.1	0.4

Table S3. Textural details of the two cements.

	Density (gcm^{-3})	BET (m^2g^{-1})	Blaine (m^2Kg^{-1})	$D_{v,10}$ (μm)	$D_{v,50}$ (μm)	$D_{v,90}$ (μm)
**PC-52.5**	3.108(1)	2.27(1)	409(8)	1.8	11.5	32.7
**PC-42.5**	3.126(1)	1.25(1)	368(1)	2.1	18.0	50.0

Table S4. Selected cumulative heat release data (from the isothermal calorimetry study) for the two employed cements. All values in J per gram of Portland cement.

	**19 h**		**47 h**		**93 h**		**7 d**		**Maximum peak**		
	**Heat/J**	**DoH$^{\$}$/%**	**Heat/J**	**DoH$^{\$}$/%**	**Heat/J**	**DoH$^{\$}$/%**	**Heat/J**	**DoH$^{\$}$/%**	**Time/h**	**Heat/J**	**DoH$^{\$}$/%**
**PC-52.5, w/c=0.40**	180.8	33.7	279.8	52.4	300.7	56.3	313.5	58.7	14.2	102.9	19.3
**PC-52.5, w/c=0.50**	157.2	29.4	306.6	57.4	346.9	64.9	365.2	68.4	15.6	102.7	19.2
**PC-42.5, w/c=0.40**	116.5	22.1	220.5	41.9	270.8	51.5	291.5	55.4	10.4	50.4	9.6
**PC-42.5, w/c=0.50**	114.4	21.7	223.3	42.4	279.8	53.1	310.0	58.9	11.0	53.4	10.2

$^{\$}$Total heat of hydration of PC52.5 as calculated in ref.[21] option-1: **534 J.**

$^{\$}$Total heat of hydration of PC42.5 as calculated in ref.[21] option-1: **526 J.**

Table S5. Rietveld quantitative phase analysis (of MoKα_1 radiation powder diffraction data) results (wt%) for the studied PC-52.5 pastes, w/c=0.40. Laboratory X-ray diffraction data were taken on the same capillary used for the laboratory, attenuation-contrast, microtomographic study. The data are referenced to 100 grams of paste.

phases	t_0^*	t=22h	t=50h	t=96h
C_3S	43.57	18.9	17.0	16.3
β-C_2S	8.43	8.3	8.7	9.1
C_3A	5.86	2.9	1.9	1.5
C_4AF	8.21	7.7	5.8	5.4
$Cc^{\#}$	2.00	3.1	3.2	3.6
CH	-	10.6	13.5	13.8
AFt	-	12.2	12.4	12.4
AFm-Hc	-		0.4	0.4
C-S-H$^{\&}$	-	20.3	20.6	20.9
Fe-Si-Hg$^{\&}$	-	0.5	2.8	3.1
FW$^{\&}$	28.57	14.4	12.9	12.7
DoH C_3S (%)	-	56	61	63
DoH C_2S (%)	-	0	0	0
DoH C_3A (%)	-	50	67	74
DoH C_4AF (%)	-	5	29	34

* This cement also has at t_0: 1.4 wt% of gypsum, 1.4 wt% of bassanite and 0.6 wt% of quartz.

& C-S-H, Fe-Si-Hg and FW (free water) contents calculated from the assumed chemical reactions as described in supplementary methods.

\# The calcite content increased from 2.0 wt% at t=0 to 3.6 wt% at 96 h, highlighting a significant carbonation of the paste within this large capillary, i.e. 1 mm of diameter. The thinner capillary used in the PXCT study, i.e. 0.2 mm of diameter, did not show a measurable conversion of CH to Cc, see below. Carbonation of a cement paste has been previously measured by PXCT, when its extension was significant.[22]

Table S6. Experimental mean β values converted to μ values obtained from a VoI in the PXCT study. The calculation of the w/c ratio of this region at the different hydration ages is also included. More details about this calculation is given in the Supplementary Methods.

Scan	Experimental β*	Experimental μ/cm^{-1}	Weight /g		Weight /wt%		w/c ratio
			water	cement	water	cement	
PXCT-19h	1.316×10^{-7}	119.1	55.0	139.4	28.3	71.7	0.39
PXCT-47h	1.265×10^{-7}	114.5	57.2	132.8	30.1	69.9	0.43
PXCT-93h	1.274×10^{-7}	115.3	56.8	133.9	29.8	70.2	0.42

*The β values have been calculated using the same volume used for the calculation of delta but excluding the regions where the electron density is smaller than 0.24 e^-Å^3, as they have been considered air porosity. It is noted that air is not included in this calculation.

Table S7. Expected (from the crystallographic data,[23,24]) mass and electron densities. The attenuation length values are calculated[$] for the employed wavelength, E=8.93 keV. The measured electron densities for selected components from the PXCT data, where relatively large volumes could be chosen, are also given.

Cement Phases	Abbrev.	Formula	Mass density /gcm^{-3}	Electron density /e$^-$Å^{-3}	Measured electron density /e$^-$Å^{-3} (within selected particles)[#]			Attenuation length /µm	µ /cm^{-1}	β
					19 h	47 h	93 h			
#0, air	-	-	~0.00	~0.00			0.00(4)			
#1, water	-	H_2O	1.00	0.33	0.31(1)			1388.6	7.2	7.96 10^{-09}
#2, ettringite	AFt	$Ca_6Al_2(SO_4)_3(OH)_{12}\cdot 26H_2O$	1.78	0.56				154.6	64.7	7.15 10^{-08}
Hemicarbo-aluminate[=]	Hc	$Ca_4Al_2(OH)_{13}(CO_3)_{0.5}\cdot 5.5H_2O$	1.90	0.59				118.6	84.3	9.32 10^{-08}
Monosulfate[=]	AFm-SO_4	$Ca_4Al_2(OH)_{12}(SO_4)\cdot 6H_2O$	2.02	0.63				112.4	89.0	9.83 10^{-08}
#3, calcium silicate hydrate	C-S-H	$(CaO)_{1.8}(SiO_2)(H_2O)_4$	*2.11*[%]	*0.66*[%]				*95.9*	*104.3*	*1.15* 10^{-07}
#4, portlandite	CH	$Ca(OH)_2$	2.23	0.69	0.62(2)	0.649(6)	0.651(5)	61.5	162.6	1.80 10^{-07}
#5, calcium carbonate	Cc	$CaCO_3$	2.71	0.82	0.782(3)	0.776(3)	0.776(3)	66.3	150.8	1.67 10^{-07}
#6, tricalcium aluminate	C_3A	$Ca_3Al_2O_6$	3.05	0.91				49.0	204.0	2.25 10^{-07}
#7, alite	C_3S	Ca_3SiO_5	3.15	0.95	0.936(2)	0.931(2)	0.932(1)	41.8	239.1	2.64 10^{-07}
#8, belite	C_2S	Ca_2SiO_4	3.30	0.99	0.98(2)	0.98(1)	0.98(1)	43.3	231.1	2.55 10^{-07}
#9, ferrite	C_4AF	Ca_2AlFeO_5[&]	3.73	1.10				26.4	379.4	4.19 10^{-07}

[#] Electron densities, from particle picking, were obtained by the average of 10 cubes for the capillary; 5, 4, 5 and 6 grains for portlandite, calcium carbonate, alite and belite, respectively. Moreover, 5 cubes at 19 h gave the reported measured electron density for capillary water. Similarly, 5 cubes at 93 h were computed to obtain the value for air. Finally, the electron density of LDH (low density hydrates) was measured at 93 h in 5 cubes yielding 0.56(1) e$^-$Å^{-3} that it corresponds to ettringite and/or C-S-H as they cannot be distinguished.

[=] The expected values for these phases, i.e. hemicarbo-aluminate and monosulfate, are given for the sake of completeness but they are not numbered as they were not identified in the bivariate plots, see Figures S9.

[%] There are not expected values for an amorphous material. The quoted values (italics) were determined for five months cured C-S-H gel by PXCT[20].

[&] The reported values are for stoichiometric Ca_2AlFeO_5, i.e. an Al/Fe molar ratio of 1.0, which is an approximation as this ratio could be different from 1.0.

[$] The *attenuation length* values have been calculated from[25], https://henke.lbl.gov/optical_constants/atten2.html

The µ values were calculated from: $\mu\ [cm^{-1}] = \frac{1}{Attenuation\ Length\ [\mu m] \times 10^{-4}}$

Finally, β was calculated as $\beta = \frac{\mu\lambda}{4\pi}$

Table S8. Component segmentation (vol%) and average electron densities obtained by PXCT at the different hydration ages; expected electron densities (from crystallographic data when it is possible) are also given for reference.

Component	Expected electron density /$e^{-}Å^{-3}$	19 h			47 h			93 h		
		Vol /%	Electron density* /$e^{-}Å^{-3}$	Electron density& /$e^{-}Å^{-3}$	Vol /%	Electron density* /$e^{-}Å^{-3}$	Electron density& /$e^{-}Å^{-3}$	Vol /%	Electron density* /$e^{-}Å^{-3}$	Electron density& /$e^{-}Å^{-3}$
Capillary	-	-	-	0.63(1)	-	-	0.63(1)	-	-	0.63(1)
Air porosity	0.00	0.2	0.01(5)	-	4.1	0.10(1)	-	6.7	0.10(1)	-
Water porosity	0.33	15.1	0.33(6)	-	2.2	0.33(5)	-	2.8	0.32(6)	-
LD-Hydrates	*0.38-0.53*	45.5	0.50(4)	-	56.5	0.52(4)	-	51.8	0.53(4)	-
Portlandite	0.69	4.6	0.63(1)	0.62(2)	13.1	0.62(4)	0.65(1)	15.7	0.62(4)	0.65(1)
Calcite	0.82	2.5	0.74(2)	0.78(1)	2.2	0.74(4)	0.78(1)	2.3	0.74(5)	0.78(1)
Belite/Alite/C_3A	0.99/0.95/0.91	30.4	0.90(1)	0.94(1)/0.98(2)	20.3	0.90(4)	0.93(1)/0.98(1)	19.3	0.90(5)	0.93(1)/0.98(1)
Ferrite	1.10	2.1	1.02(2)	-	1.5	1.01(4)	-	1.4	1.02(4)	-

*Electron densities, from full volume, were obtained by segmentation excluding the external voxels to avoid partial volume effect

&Electron densities, from particle picking, were obtained by the average of 10 cubes for the capillary; 5, 4, 5 and 6 grains for portlandite, calcium carbonate, alite and belite, respectively.

Table S9. Volume percentages for the cement pastes at the different ages of hydration determined by the techniques used in this paper. Degrees of hydration are also included.

Technique	Hydration age /h	Porosity /vol%: air & water	LD-hydrates /vol%	Portlandite & calcite /vol %	Anhydrous components /vol %: $C_3S/C_2S/C_3A/C_4AF$	DoH /%
Calorimetry PC-52.5, w/c=0.40	19	-	-	-	-	34
	22	-	-	-	-	39
	47-50	-	-	-	-	53
	93-96	-	-	-	-	56
Calorimetry PC-42.5, w/c=0.50	19	-	-	-	-	22
	47	-	-	-	-	42
	93	-	-	-	-	53
LXRPD PC-52.5, w/c=0.40	0	56.1$	-	-	39.9$	-
	22	26.4	39.6	11.6	22.0	45
	50	22.3	44.8	13.7	20.6	48
	96	21.9	45.5	14.2	19.9	50
Lab-µCT PC-52.5, w/c=0.40	0	56.1$			39.9$	-
	19	5.6	35.7	35.7	23.5	41
	47	2.1	21.7	60.8	15.4	61
	93	2.1	20.2	62.8	15.0	62
Syn-µCT PC-42.5, w/c=0.50	0	61.5#	-	-	35.0#	-
	19	1.9	43.8	27.3	27.0	23
	47	14.5	32.9	31.7	20.8	41
	93	13.7	34.3	35.2	16.9	52
PXCT PC-52.5, w/c=0.40	0	56.1$			39.9$	-
	19	15.3	45.5	7.1	32.1	20
	47	6.3	56.5	15.3	21.8	45
	93	9.5	51.8	18.0	20.7	48

$ The amount of water and clinker phases is 96.0 vol%. The remaining 4.0 vol% is due to the minor components: gypsum, bassanite, calcite and quartz

The amount of water and clinker phases is 96.4 vol%. The remaining 3.6 vol% is due to the minor components: gypsum, bassanite, calcite and quartz

• Supplementary Figures

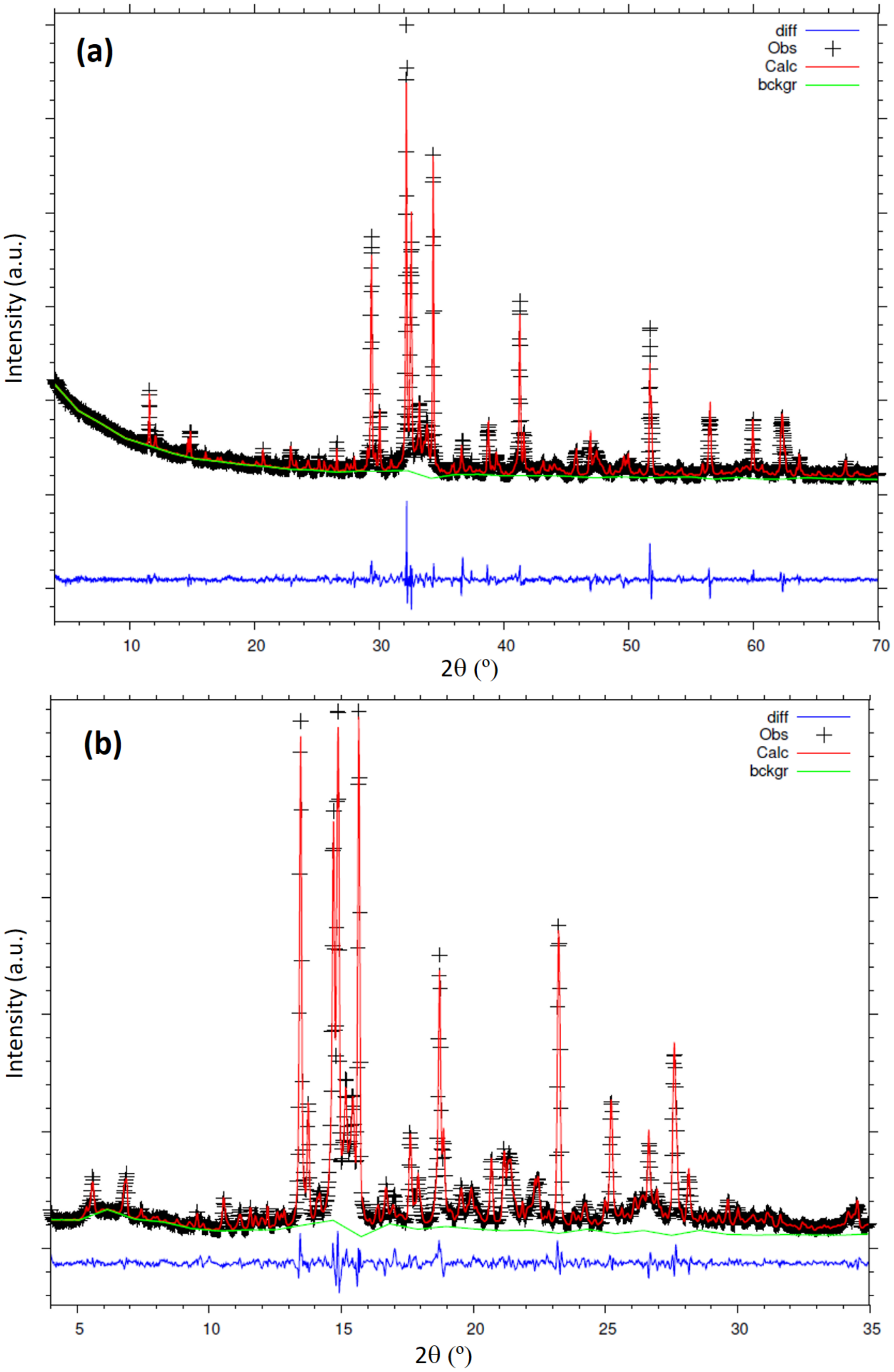

Figure S1. Laboratory Rietveld plots for the anhydrous cements. (a) PC-52.5 (CuKα_1 radiation, λ=1.5416 Å). (b) PC-42.5 (MoKα_1 radiation, λ=0.7093 Å).

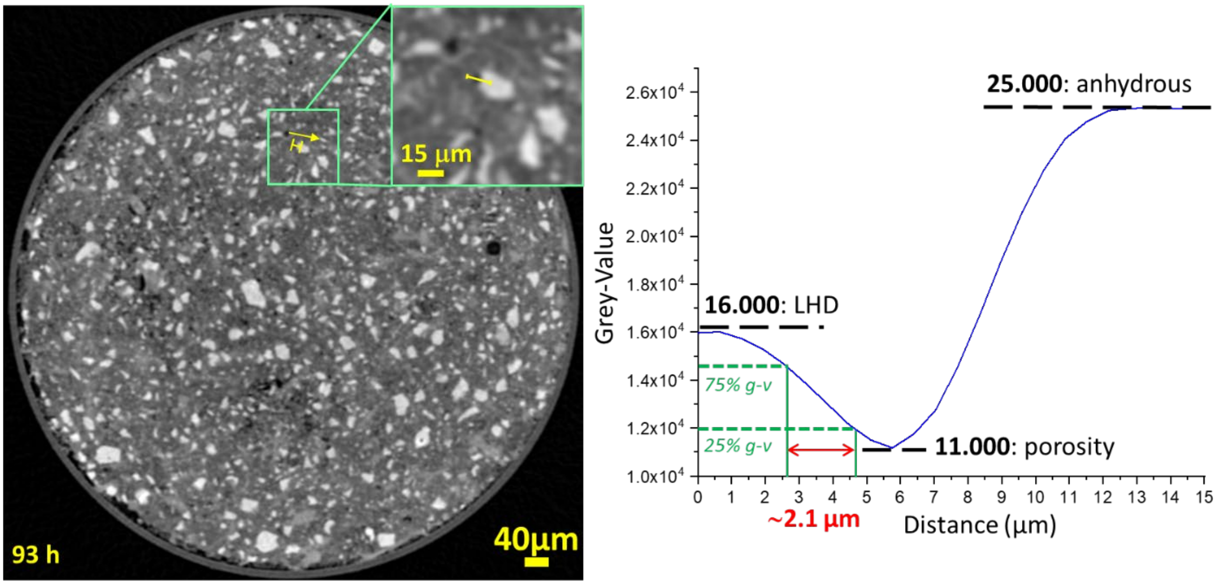

Figure S2. (Left) Selected orthoslice and (Right) grey-value profile of the yellow line (shown in the right panel) including a sharp interface for the laboratory, attenuation-contrast, microtomographic study (PC-52.5 paste with w/c=0.40, dataset at 4 days of hydration). The estimated spatial resolution, from this approach, is ~1.9 μm.

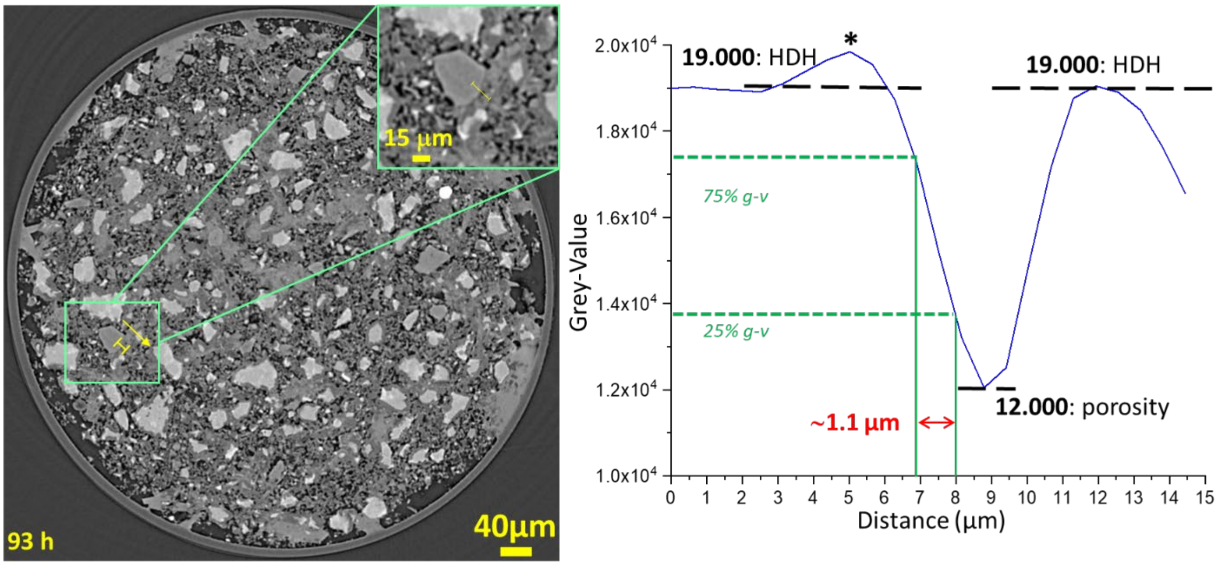

Figure S3. (Left) Selected orthoslice and (Right) grey-value line profile of a sharp interface for the synchrotron, phase propagation based-contrast, microtomographic study (PC-42.5 paste with w/c=0.50, dataset at 4 days of hydration). The estimated spatial resolution, from this approach, is ~ 0.75 μm. The star symbol highlights the small artefact (edge enhancement not fully corrected by the Paganin algorithm) which is commonly observed in in-line propagation-based phase-contrast synchrotron tomography.

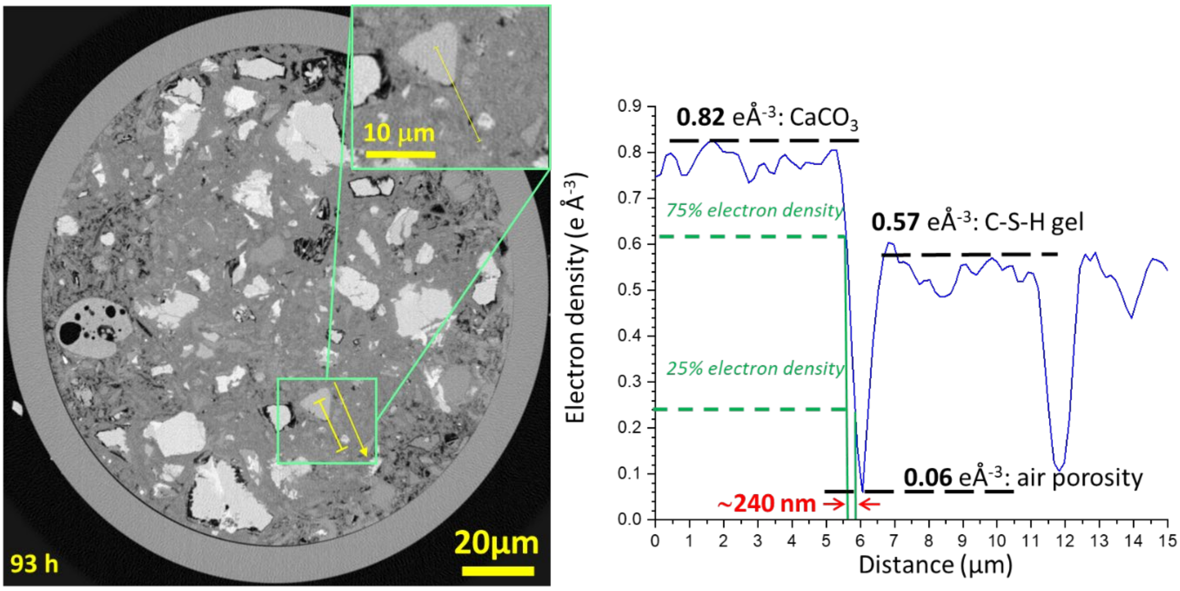

Figure S4. (Left) Selected orthoslice and (Right) line profile of a sharp interface for the near-field ptychographic X-ray computed tomographic study (PC-52.5 paste with w/c=0.40, dataset at 4 days of hydration). The estimated spatial resolution, from this approach, is ~250 nm.

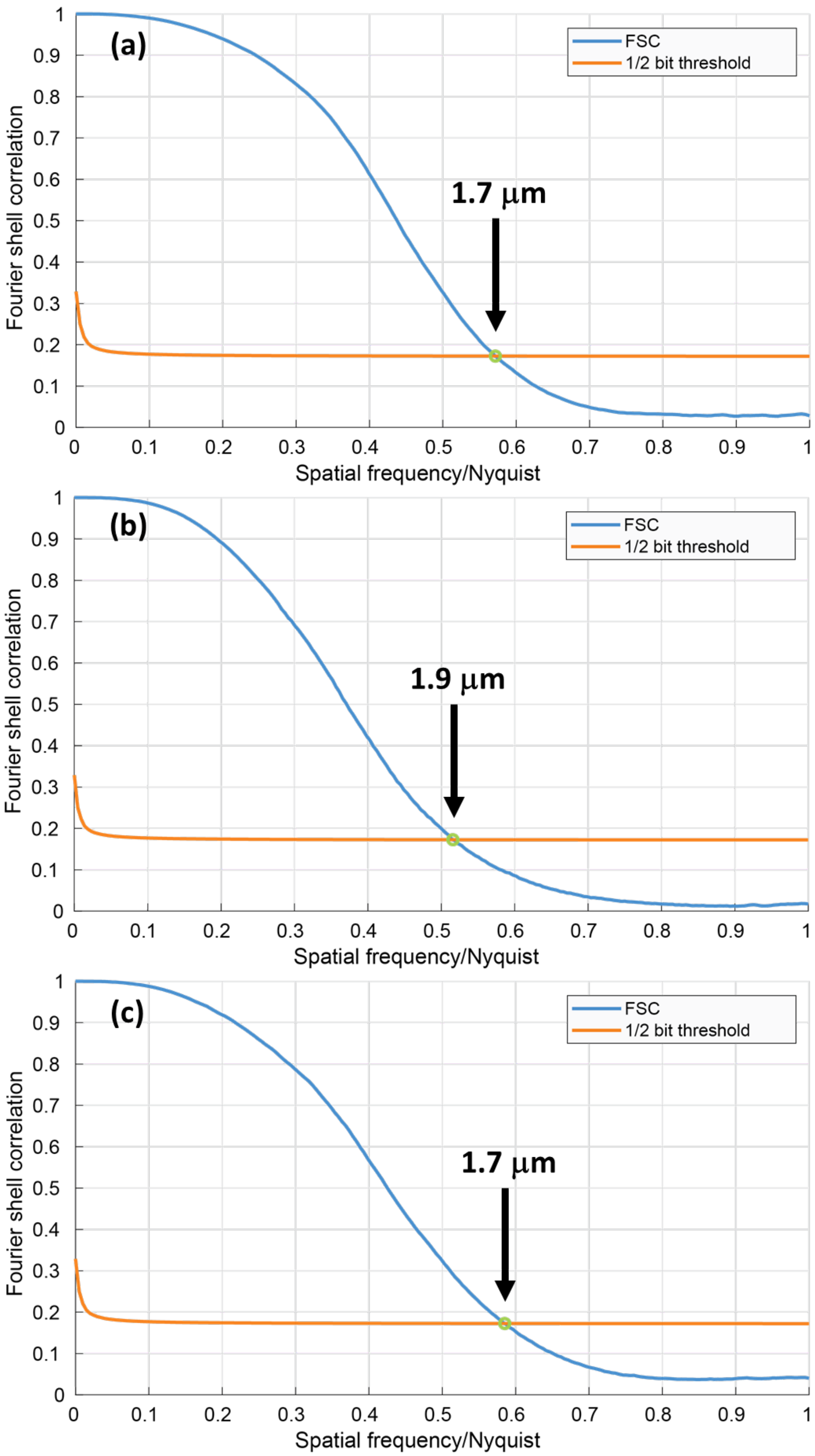

Figure S5. Fourier Shell Correlation plots for the laboratory, attenuation-contrast, microtomographic study, PC-52.5 paste (w/c=0.40) at (a) 19 h, (b) 47 h and (c) 93 h of hydration. The cuts between the FSC traces and the threshold lines give an indication of the spatial resolution of each tomogram.

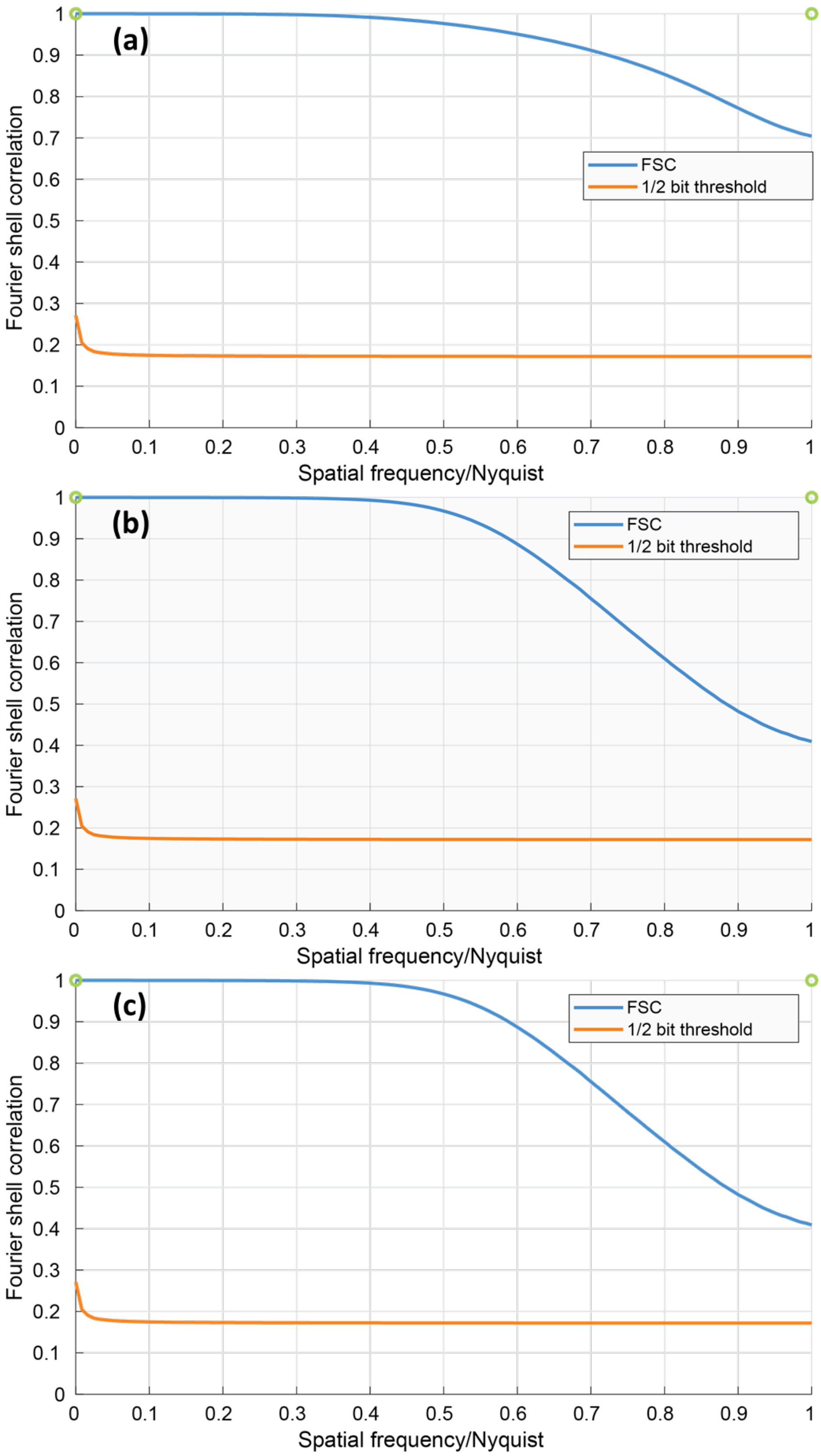

Figure S6. Fourier Shell Correlation plots for the propagation-based synchrotron phase-contrast X-ray computed microtomographic study, PC-42.5 paste (w/c=0.50) at (a) 19 h, (b) 47 h and (c) 93 h of hydration. The FSC traces do not cut the threshold indicating that, from this approach, the overall spatial resolution is limited by the sampling (pixel size).

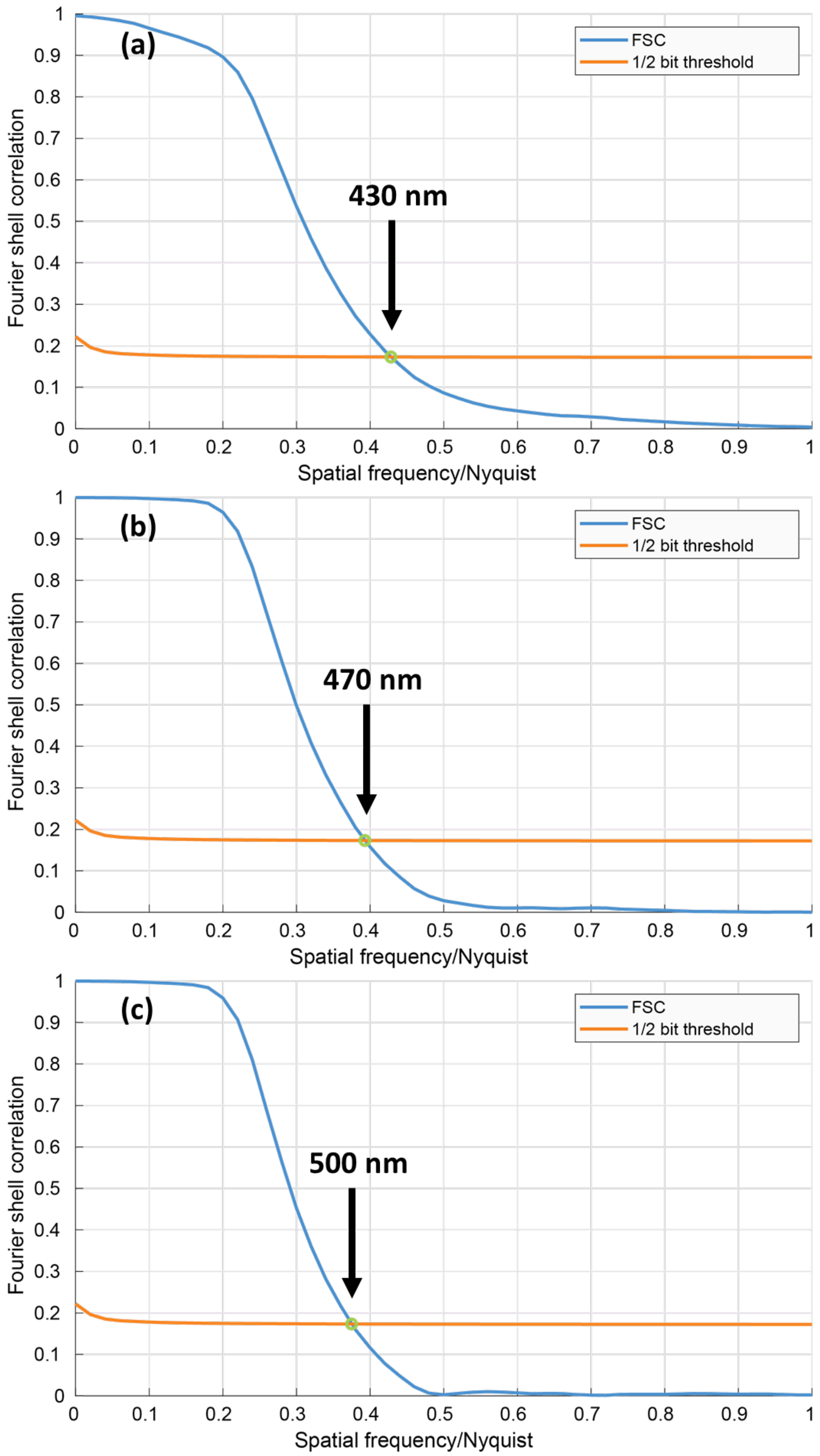

Figure S7. Fourier Shell Correlation plots from the near-field ptychographic X-ray computed tomographic study for the PC-52.5 paste (w/c=0.40) at (a) 19 h, (b) 47 h and (c) 93 h of hydration. The cuts between the FSC traces and the threshold lines give an indication of the spatial resolution of each tomogram. The slightly better spatial resolution measured for the 19 h tomogram is very likely due to the smaller scanning step size, i.e. 6 μm, and the corresponding larger acquisition time, i.e. 3h 55 min. The scanning step size for the other two tomograms was 7 μm, yielding 3h 6 min of acquisition time.

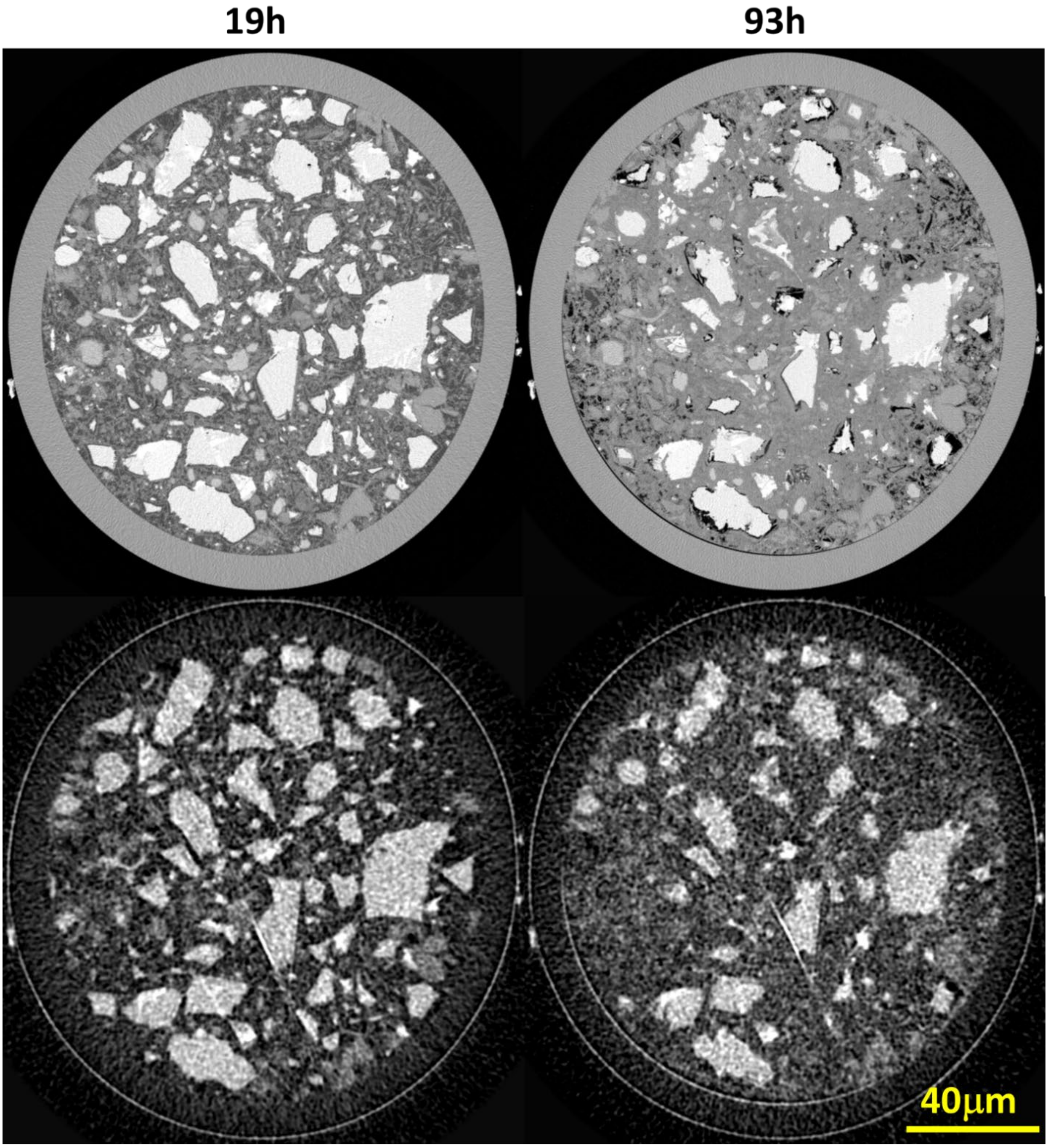

Figure S8. Selected PXCT orthoslices at 19 and 93 h of hydration. (Top) Electron density datasets. (Bottom) Absorption datasets.

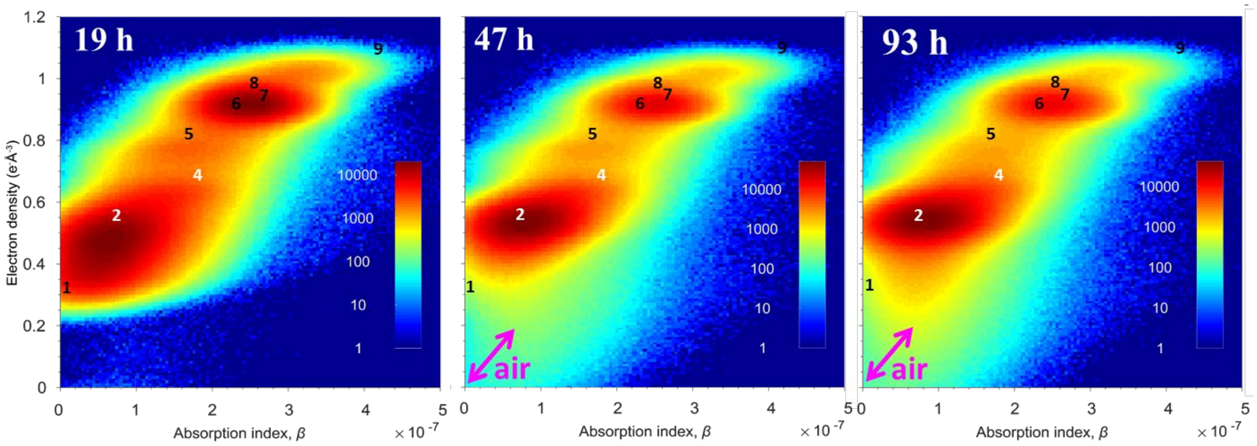

Figure S9. Bivariate histograms of electron densities and absorption indexes (β) for the PXCT study of the PC-52.5 paste (w/c=0.40) at the three studies hydration ages. The positions of the different components, as expected from the crystallographic data, are given. C-S-H gel position (component #3) is not given, as it is a non-stoichiometric solid, but it should be close to ettringite (#2). The electron density and absorption of air is zero but the partial volume effect slightly displaces the values, see pink arrows. For the numbers/components, the reader is referred to Table S7.

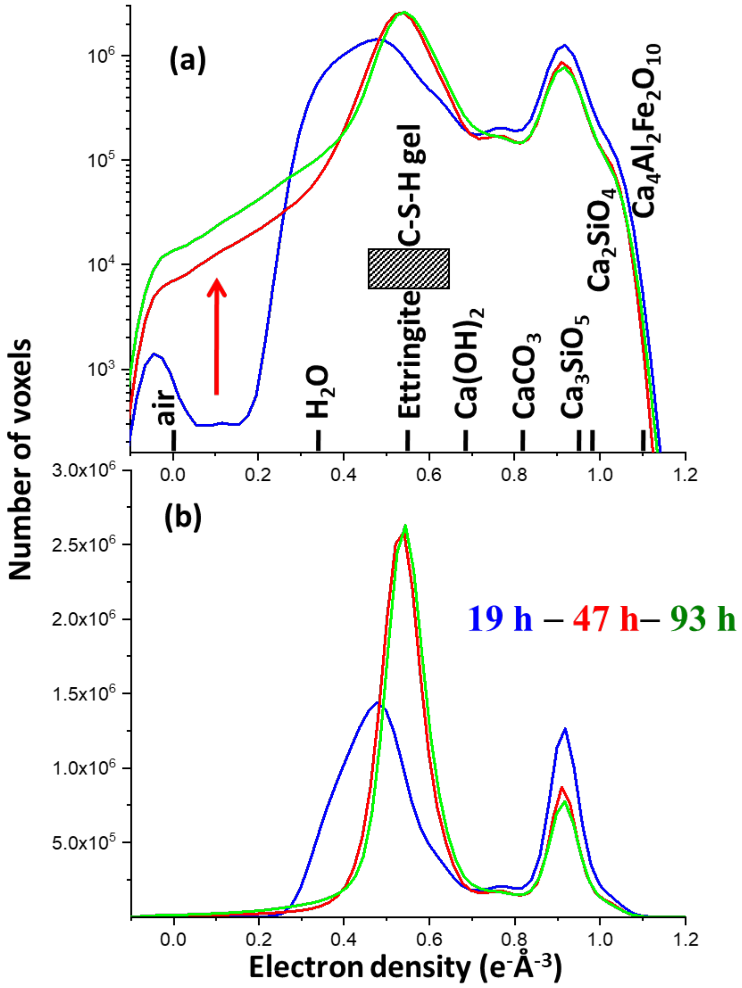

Figure S10. Volume-of-interest histogram of the electron densities for the PXCT study of the PC-52.5 paste (w/c=0.40) at (blue) 19 h, (green) 47 h and (red) 93 h of hydration. (a) Logarithmic scale, (b) Linear scale. The expected (from the crystal structures) electron densities for the different components are labelled in (a). C-S-H gel is an amorphous solid with variable water content and Ca/Si molar ratio and therefore, the expected electron densities are a range.

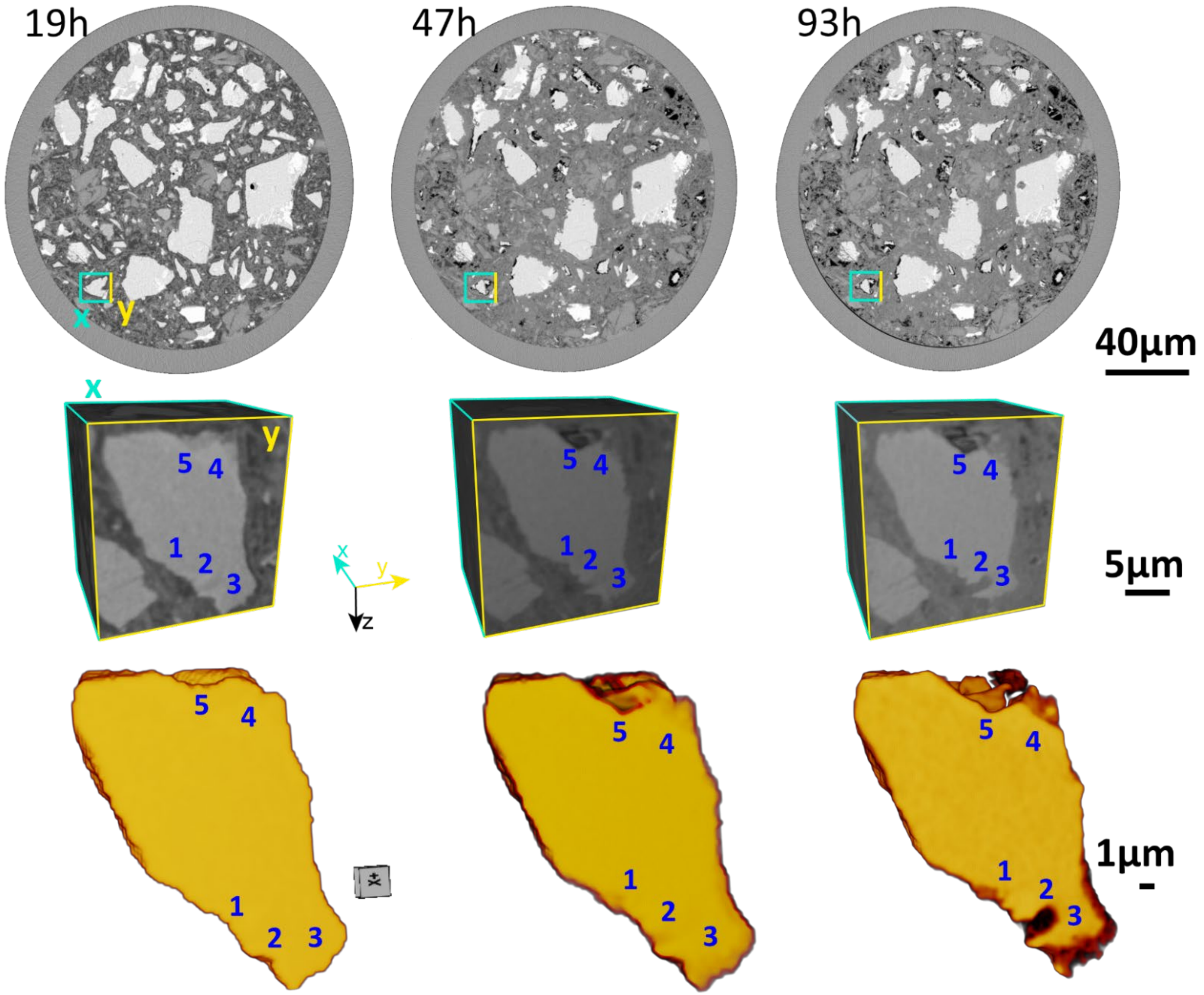

Figure S11. Second etch-pit evolution picture. (Top row) PXCT orthoslices at the three studied ages. (Intermediate) 3D rendering of a volume including a fraction of the alite particle highlighted in the top panels. (Bottom) 3D representation of the segmented particle to highlight the evolution of the etch-pits. These 3D rendered views do not show exactly the electron densities as they are affected by visualization features like the lighting source.

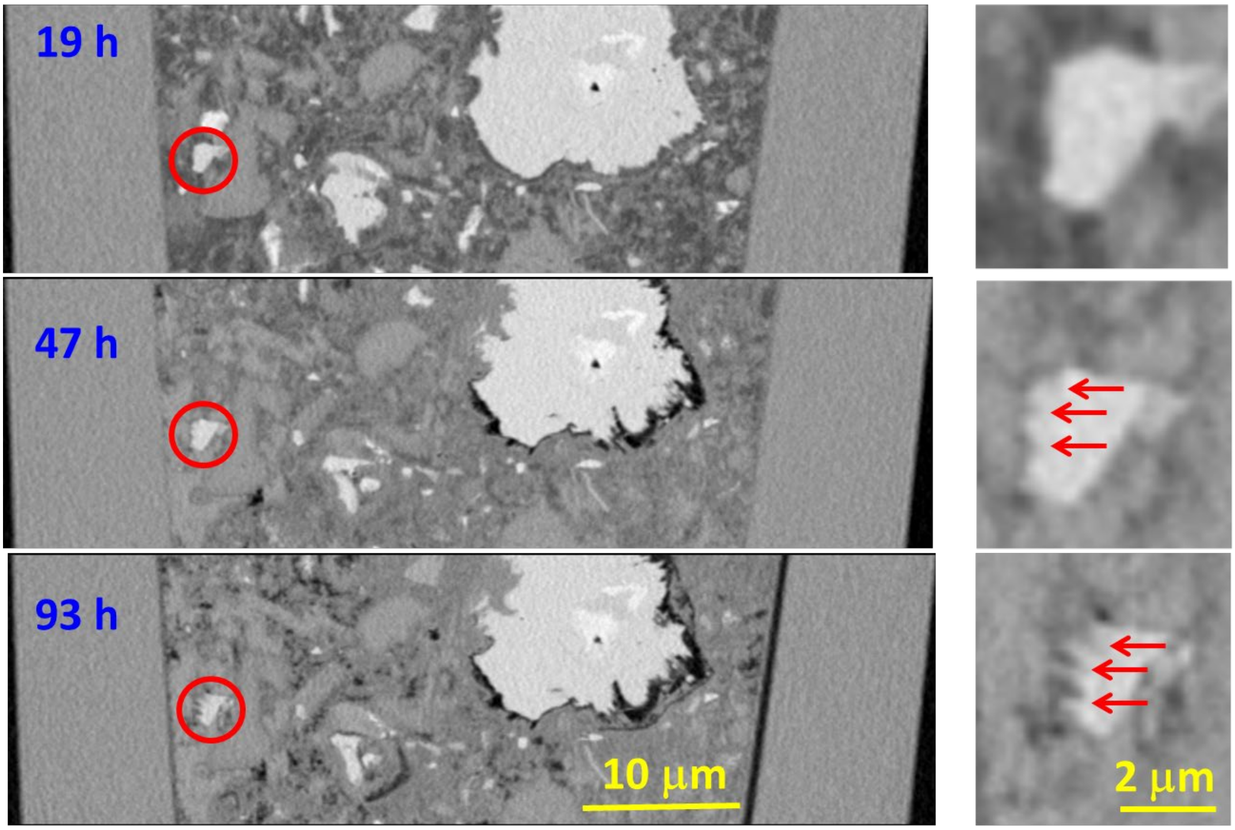

Figure S12. Selected PXCT vertical views at the studied ages showing the evolution of the PC-52.5 paste. This series is intended to show the evolution of water porosity (dark-grey) towards air porosity (black) with time. Moreover, the enlarged views (right images) show the evolution of a small alite particle, initial size about 3 µm, which develops etch-pits of sizes of ~700 nm, highlighted with red arrows.

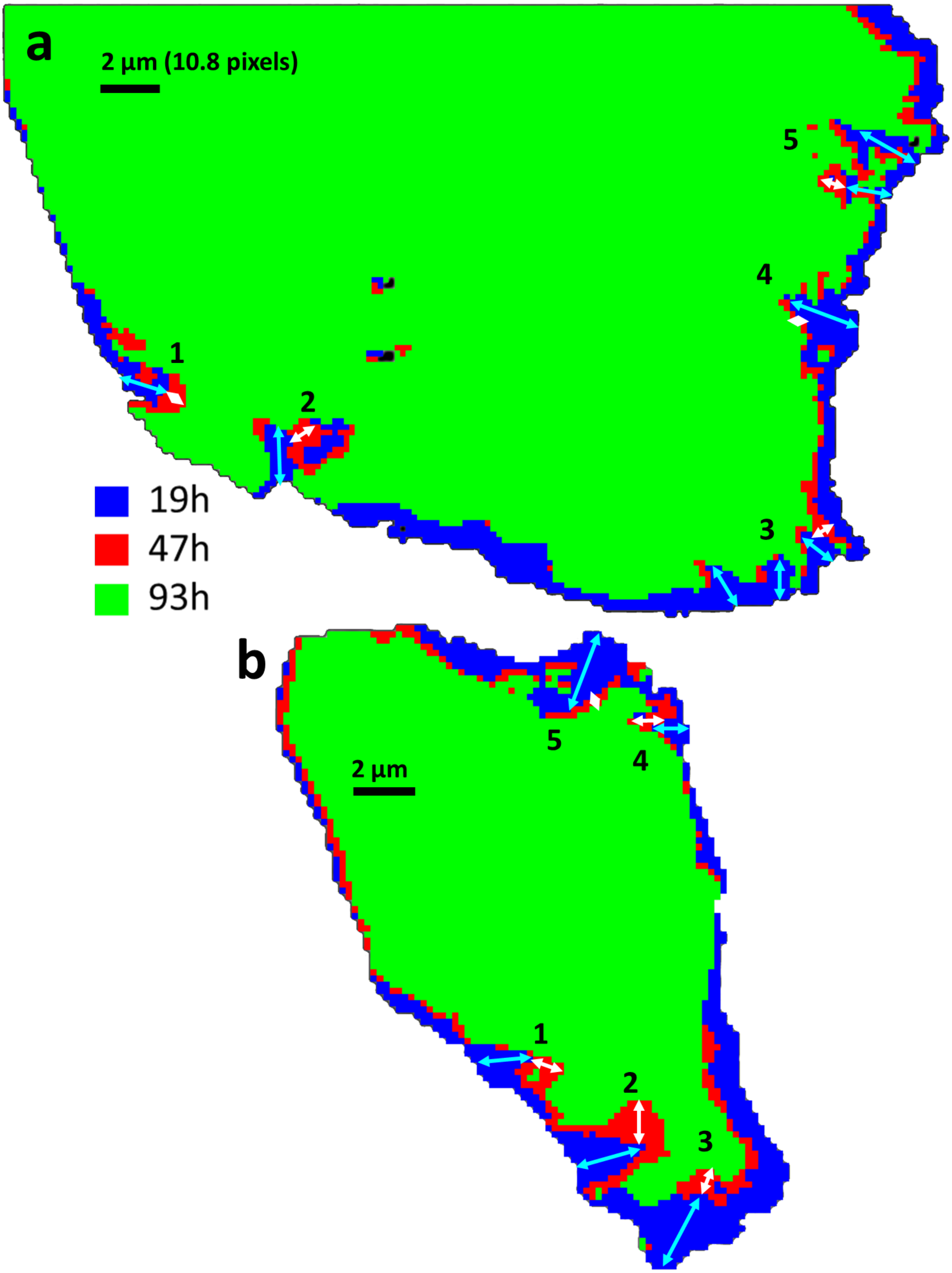

Figure S13. Overlay of the (2D-projected) alite segmented pixels during the hydration process to show the large variability in the growth rates of the etch-pits. a, five etch-pits corresponding to the Figure 4 of the manuscript. b, five etch-pits corresponding to the Figure S11 of this SI. Pale-blue arrows show size changes from 19h to 47h, meanwhile white arrows display to the changes from 47h to 93h.

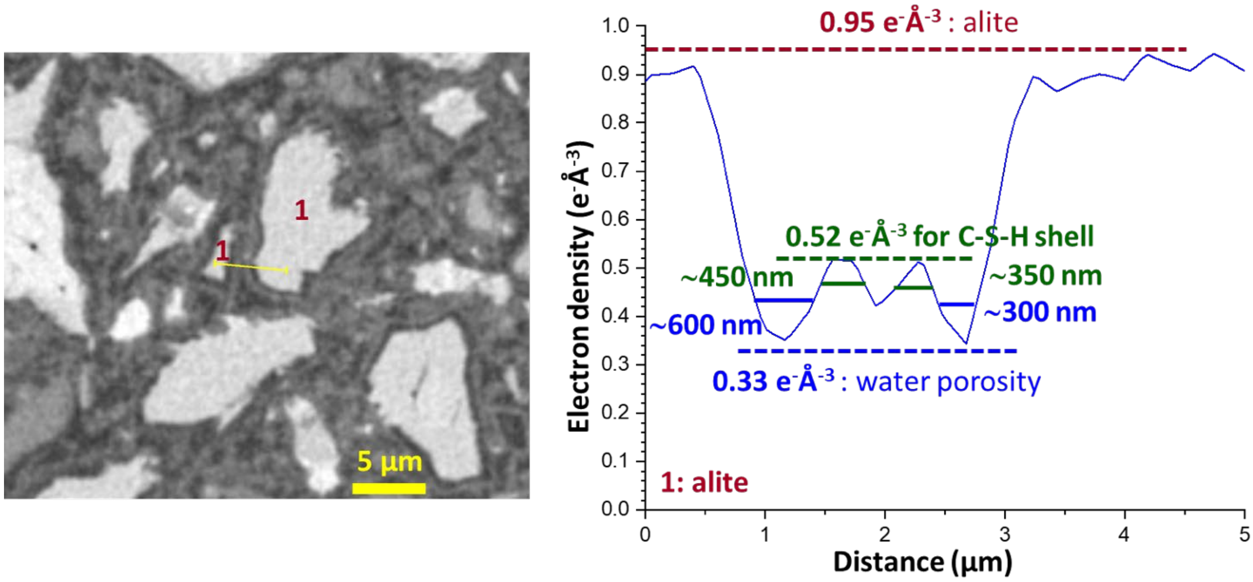

Figure S14. (Left panel) Selected 2D view of the PXCT data at 19 h. (Right) Electron density profile corresponding to the yellow straight line. The line profile signals the water porosity region (blue) surrounding the two alite particles, with the sizes of the C-S-H gel shells and their electron densities given in green.

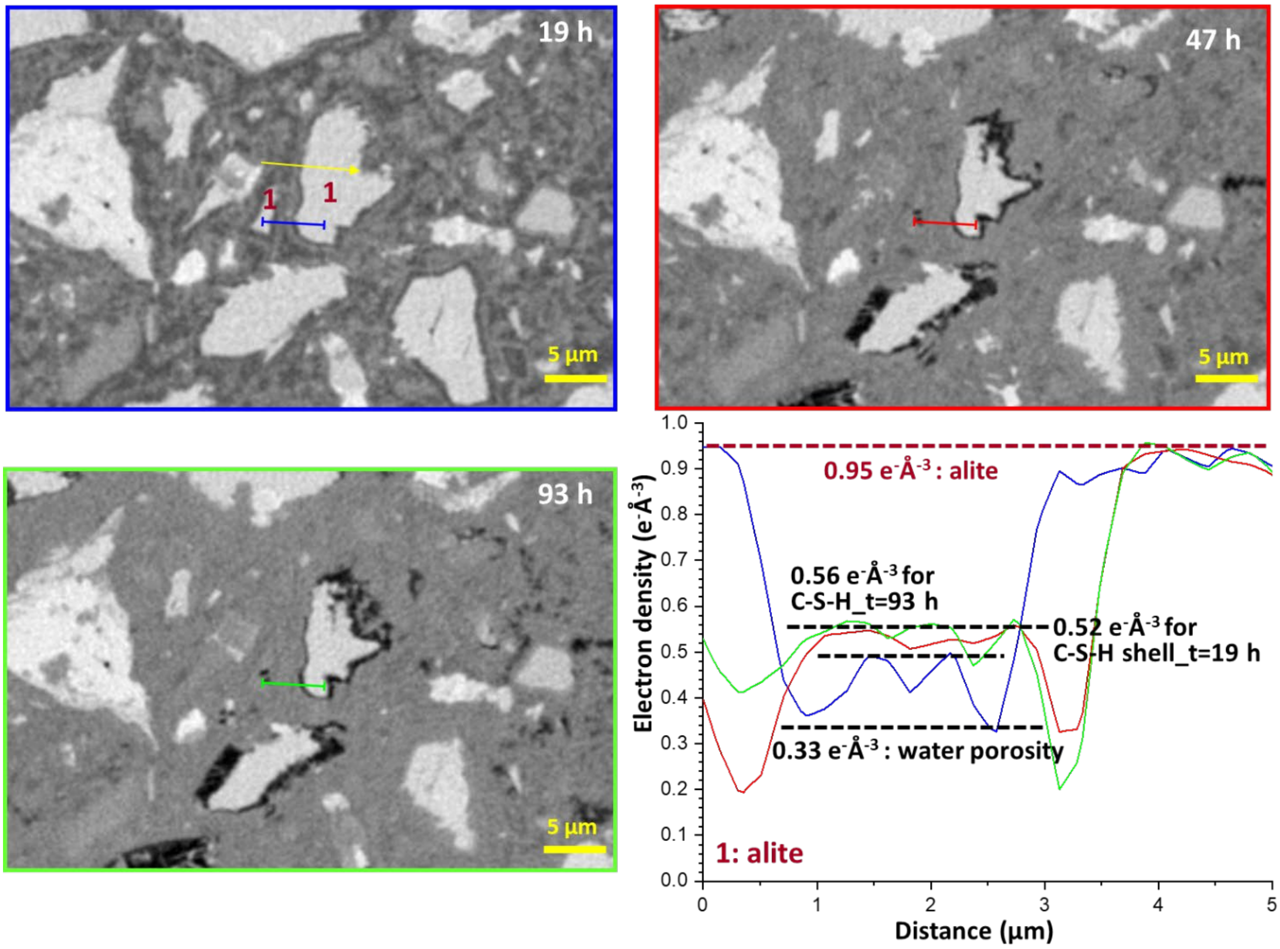

Figure S15. Study of the alite dissolution and C-S-H gel (shell) densification with hydration time. Same region than that shown in Fig. S13.

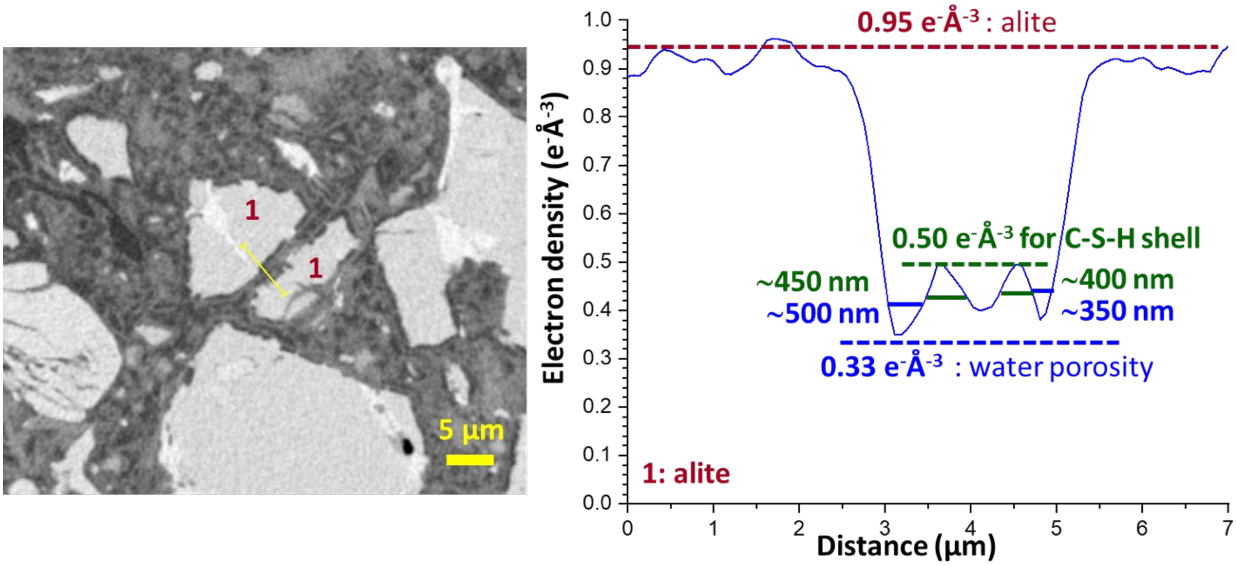

Figure S16. (Left panel) Selected 2D view of the PXCT data at 19 h. (Right) Electron density profile corresponding to the yellow straight line. The line profile signals the water porosity region (blue) surrounding the two alite particles, with the sizes of the C-S-H gel shells and their electron densities given in green.

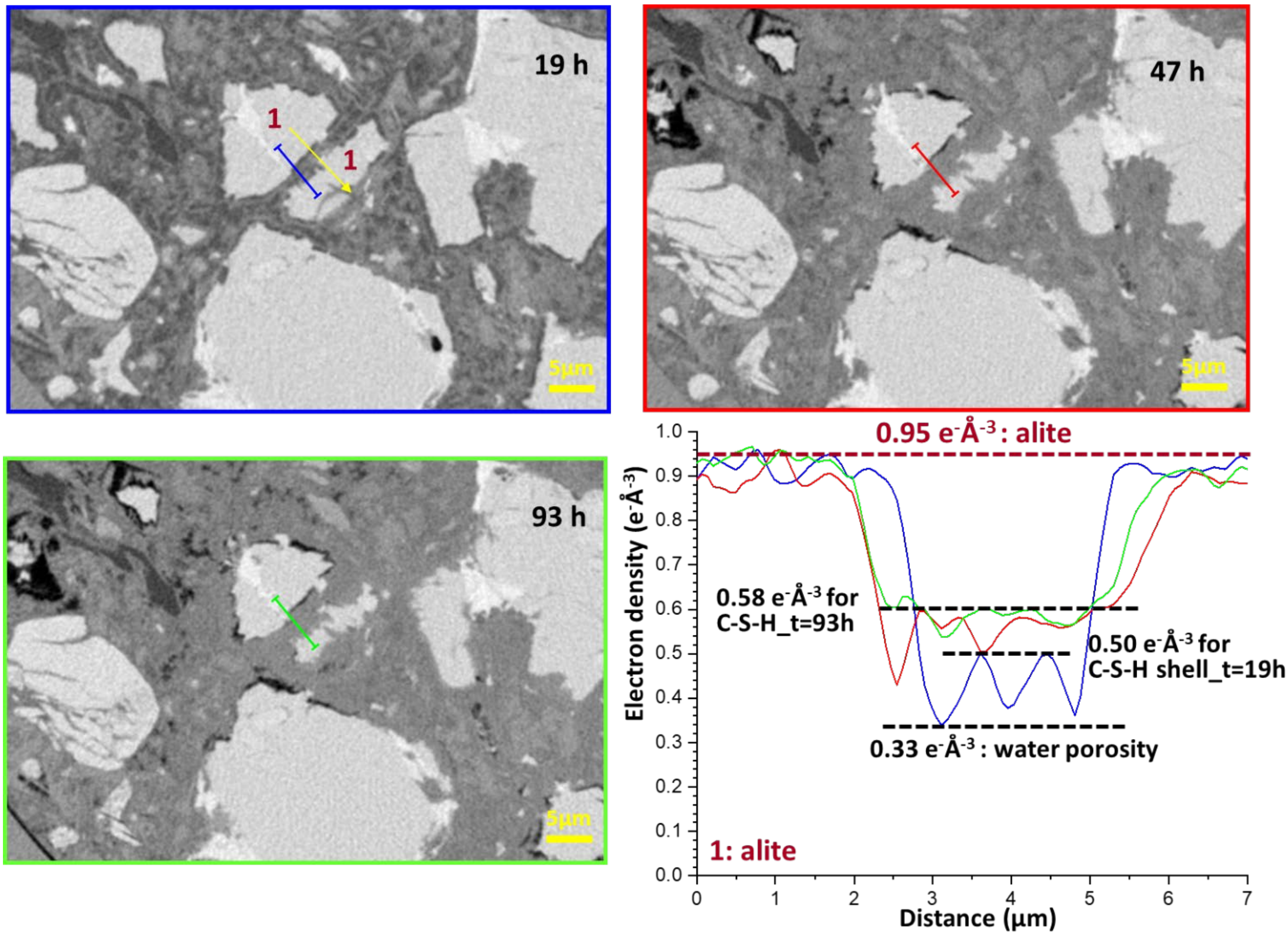

Figure S17. Study of the alite dissolution and C-S-H gel (shell) densification with hydration time. Same region than that shown in Fig. S15. Capillary pore water is still visible at 93 h in the top left region of the image.

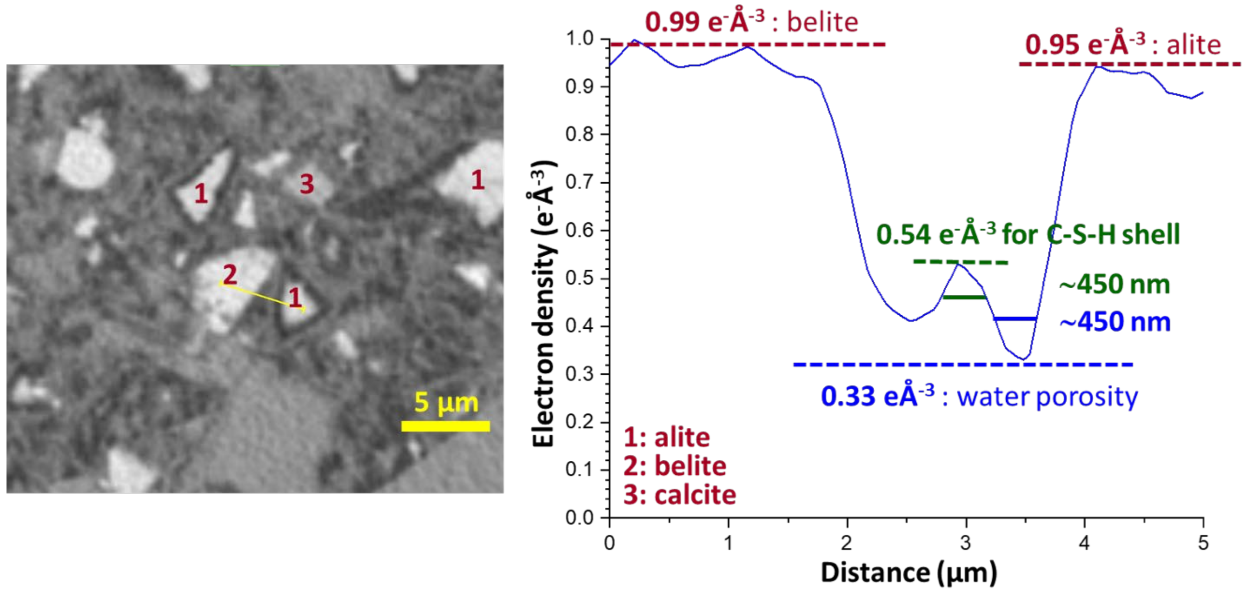

Figure S18. (Left panel) Selected 2D view of the PXCT data at 19 h. (Right) Electron density profile corresponding to the yellow straight line in the right image. The C-S-H porous shell covers every alite particle but it does not surround belite neither calcite. The line profile signals the water porosity region (blue) surrounding alite, with the size of the C-S-H gel shell and its electron density given in green.

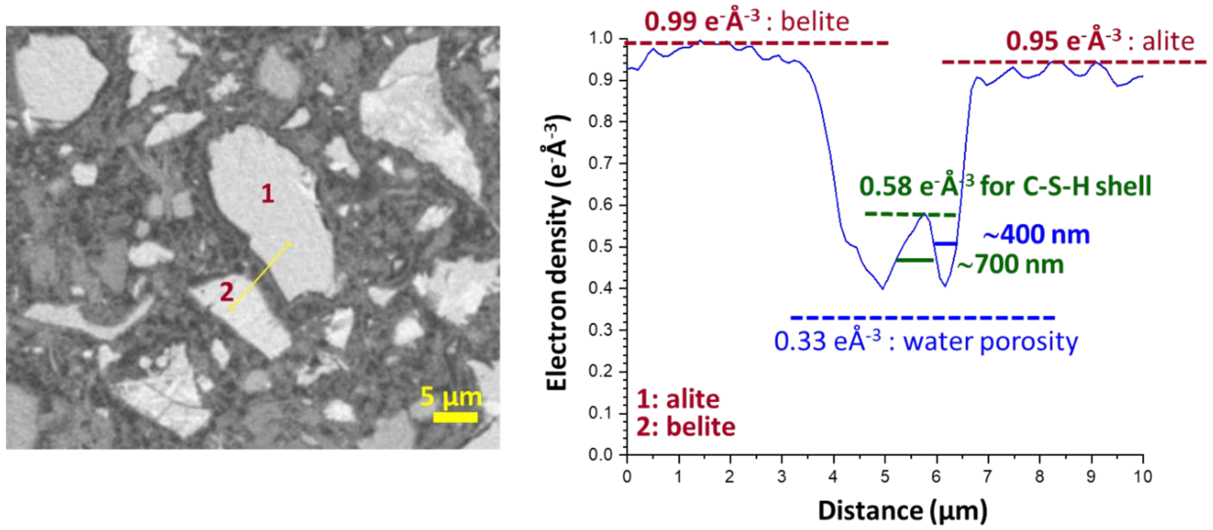

Figure S19. (Left panel) Selected 2D view of the PXCT data at 19 h. (Right) Electron density profile corresponding to the yellow straight line. The C-S-H porous shell covers every alite particle but it does not surround belite grains. The line profile signals the water porosity region (blue) surrounding alite, with the size of the C-S-H gel shell and its electron density given in green.

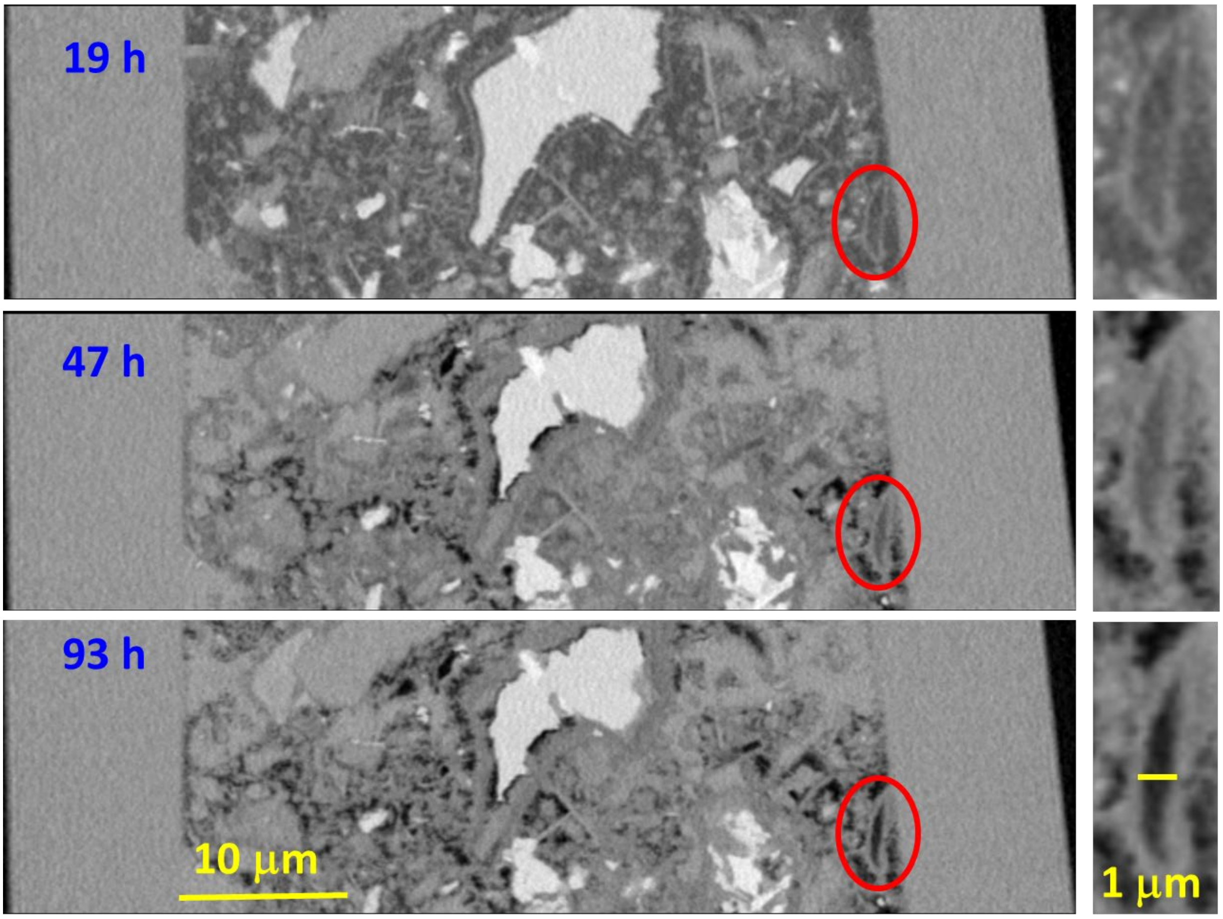

Figure S20. Selected PXCT vertical views at the studied ages showing the evolution of the PC-52.5 paste. This series is intended to show the evolution of water porosity (dark grey) towards air porosity (black) with time. The enlarged views (right images) show the change of a hollow-shell volume, also known as Hadley grain, with hydration time. The hollow-shells, Hadley grains, are fully hydrated small alite particles that contain a void within the original boundary of the anhydrous grain. The hollow regions of the Hadley grains are filled with water at 19 and 47 h but dried at 93 h, see enlarged pictures to the right. This illustrates that most of the capillary pores with sizes larger than ~1 µm are already water emptied at 93 h of hydration, see bottom right. Moreover, it also illustrated that the C-S-H shells are porous as they allow the water diffusion from the inner regions towards the exterior.

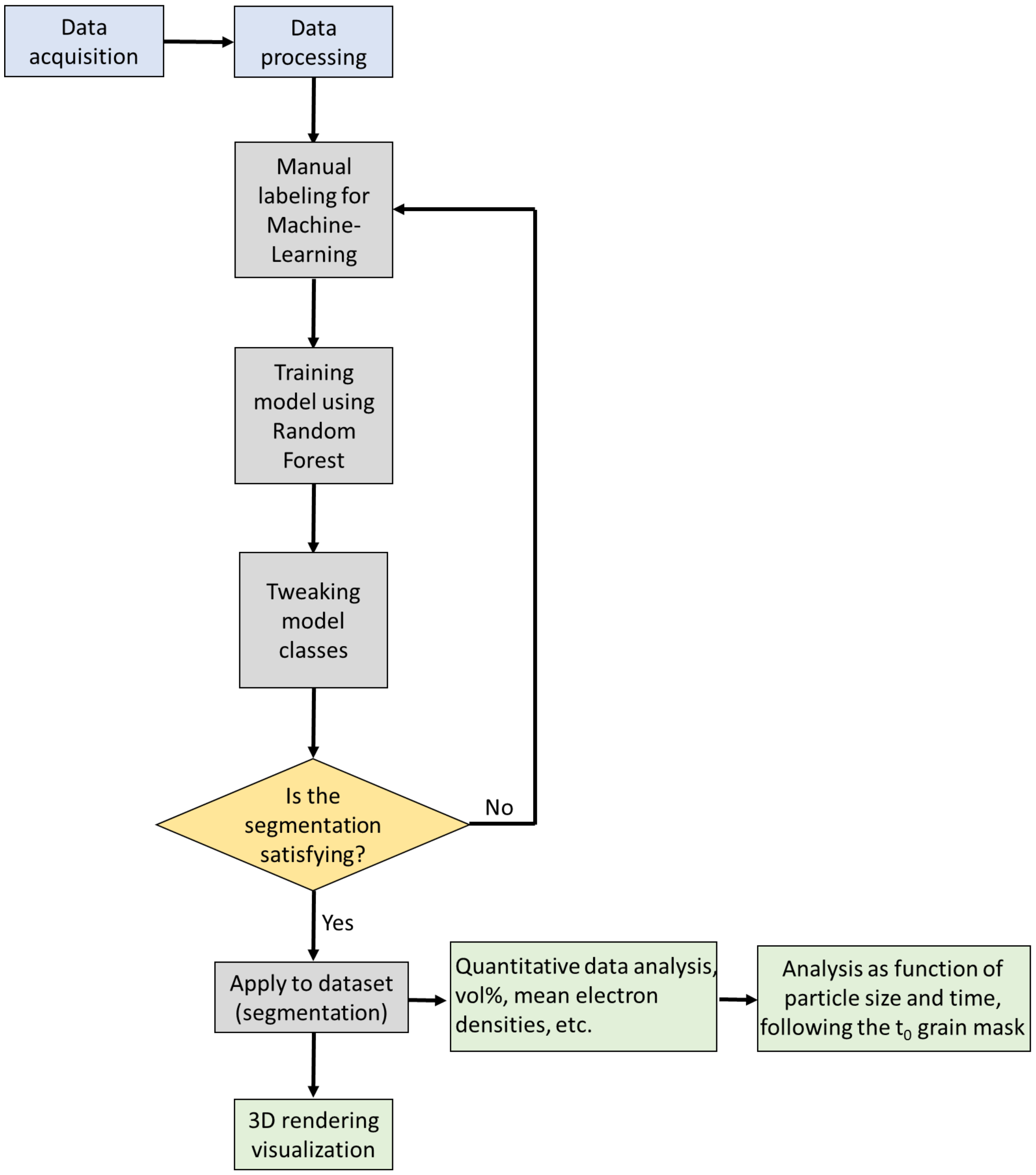

Figure S21. Flow chart describing the data treatment in this work and detailing the machine learning training steps (boxes in grey).

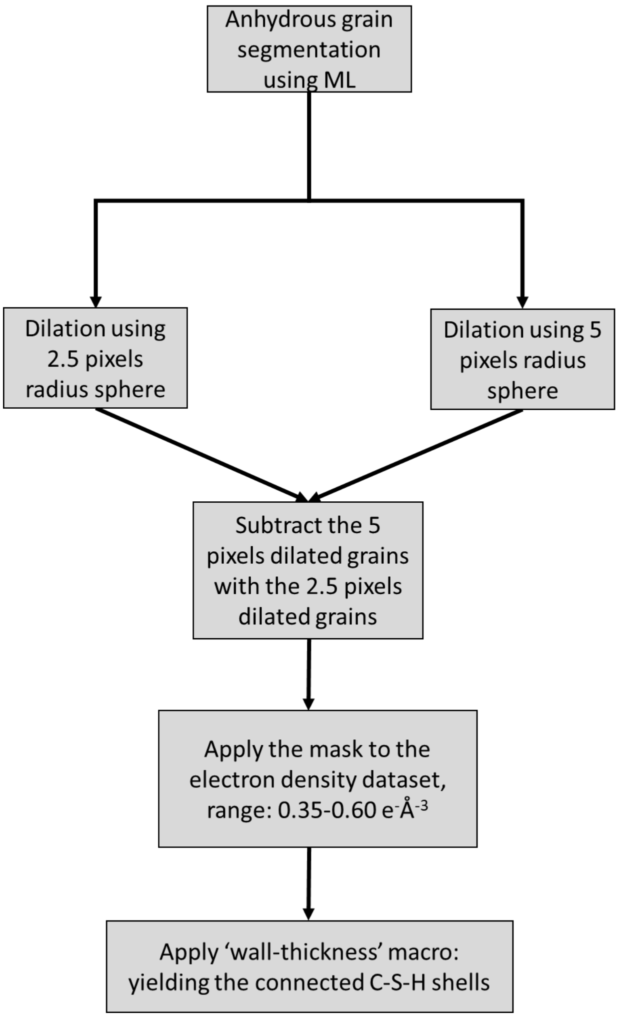

Figure S22. Flow chart detailing the steps for the C-S-H shell segmentation in the 19 h PXCT dataset.

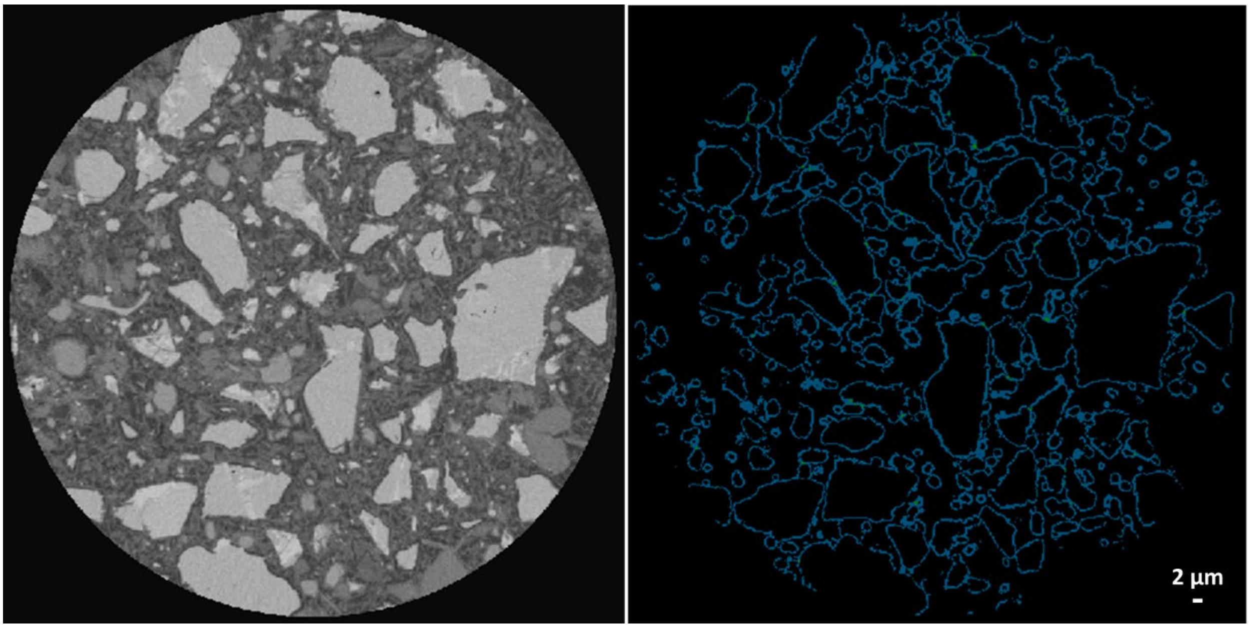

Figure S23. Views, at the same scale, comparing the C-S-H shell as observed in the PXCT raw dataset at 19 h of hydration (left) and the C-S-H shell segmentation output applying the procedure detailed in Fig. S21.

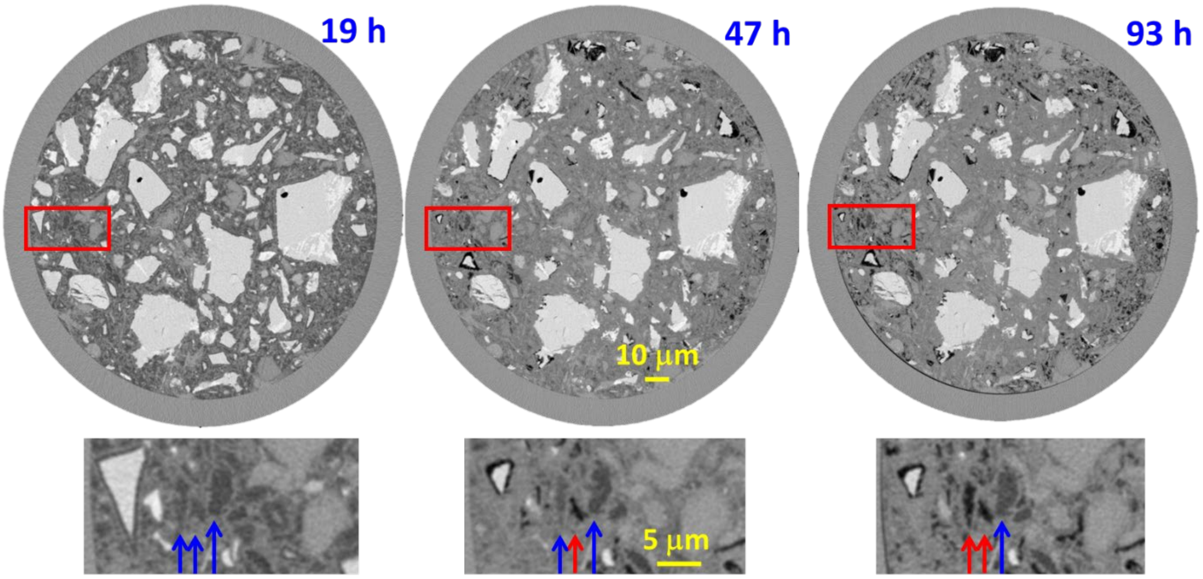

Figure S24. Selected PXCT orthoslices at the studied ages showing the evolution of the PC-52.5 paste. The enlarged views (bottom) show the evolution of porosity within the paste, where several pores of sizes smaller than ~2 µm are dried (red arrows) at 93 h but other larger, pores keep filled with water (blue arrows).

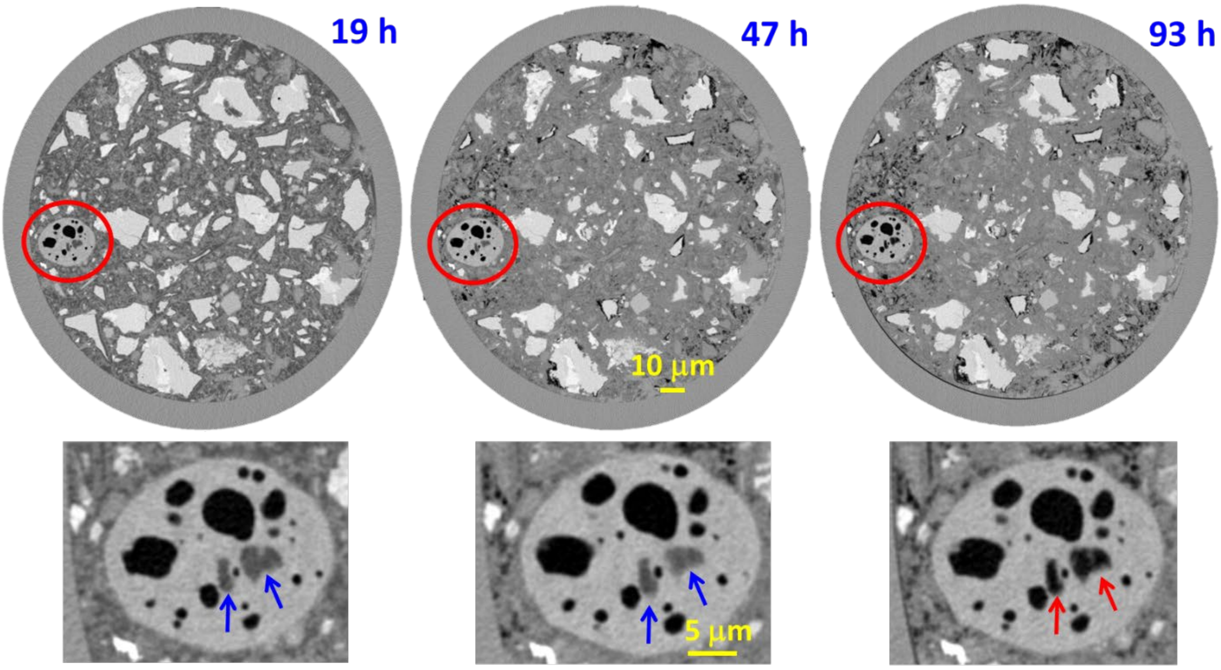

Figure S25. Selected PXCT orthoslices at the three studied ages showing the evolution of the PC-52.5 paste. The enlarged views (bottom) show the evolution of a large calcite particle with internal pores. At 19 and 47 h of hydration some pores are filled with water (highlighted with blue arrows) which are connected to the surface. At 93 h, these pores are empty (red arrows) releasing water for further hydration.

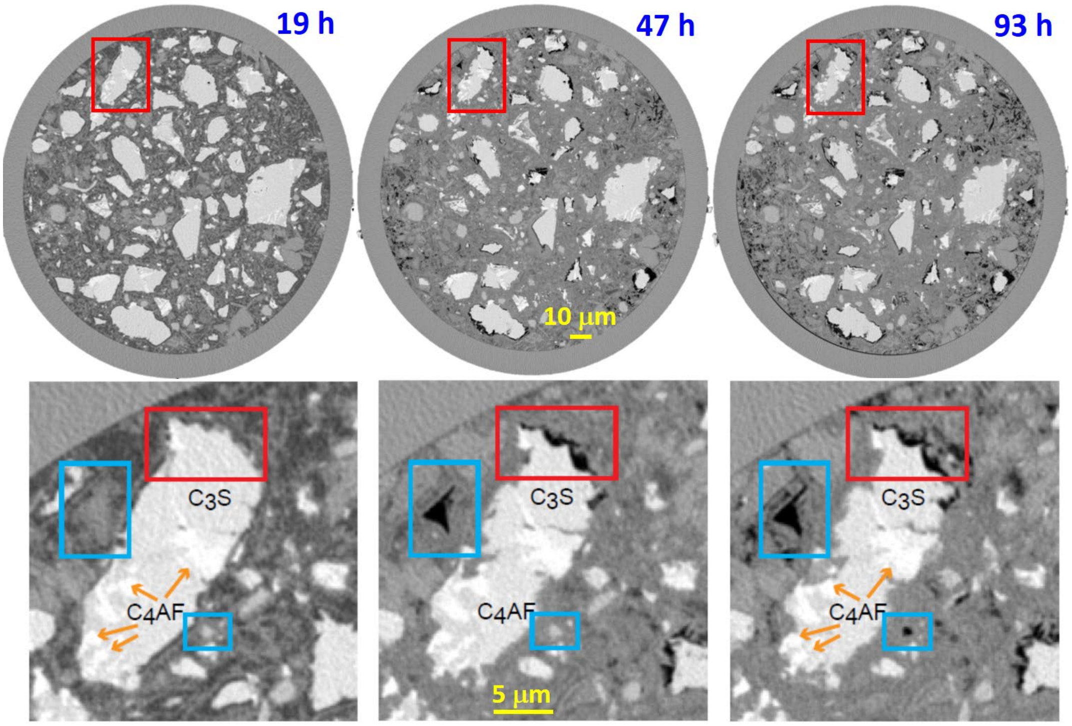

Figure S26. Selected PXCT orthoslices at the studied ages. The chemical shrinkage is evident at 93 h because the appearance of empty (black) regions. The enlarged views (bottom) show: (1) the hydration of a large alite particle with aluminoferrite, C_4AF, intergrown, i.e. the whitest regions, see brown arrows. In addition to the etch-pit evolution, it can be seen that hydration stops at the regions where C_4AF is exposed to the hydration medium; (2) the blue rectangles highlight the dissolution of C-S-H gel particles to give dry (air-filled) pores. Moreover, alite hydration also stops as soon as air porosity (pore drying) develops on the surfaces of the anhydrous grains, see red rectangles in the bottom panels.

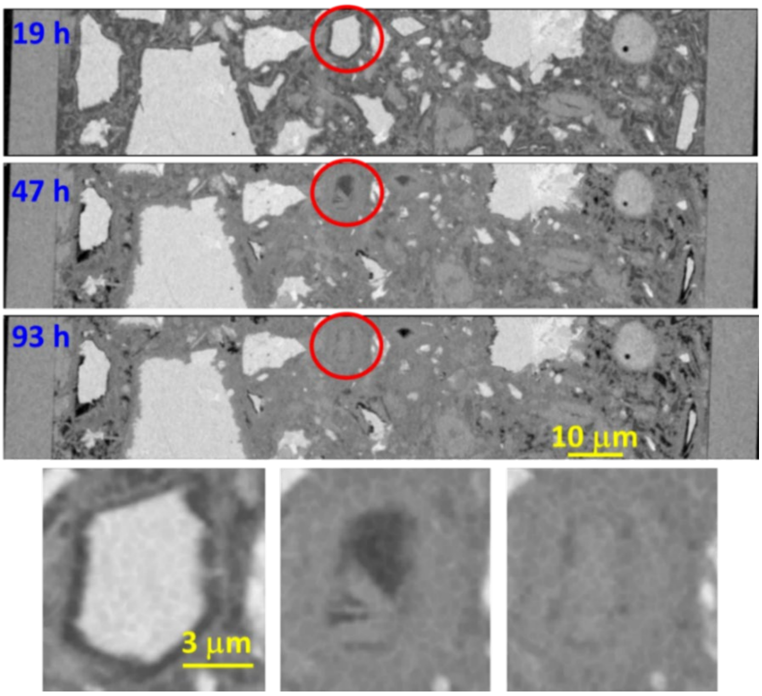

Figure S27. Selected PXCT vertical pictures with enlarged views (bottom) displaying the hydration pathway of a very fast dissolving particle, i.e. a 4 µm particle fully dissolved between 19 and 47 h. The electron density of this small volume, 0.91 $e^{-}Å^{-3}$, is compatible with C_3S or C_3A. It already shows a gap at 19 h indicating a highly soluble component. At 47 h, two hydrate rods of diameter smaller than 1 µm, morphologically suggesting ettringite, grow in the pristine region which is filled with capillary water. At 93 h, the volume is fully occupied by hydrate(s). The dissolution rate between 19 and 43 h is faster than 75 nm/h suggesting C_3A. However, the chemical nature of this tine particle could not be firmly established.

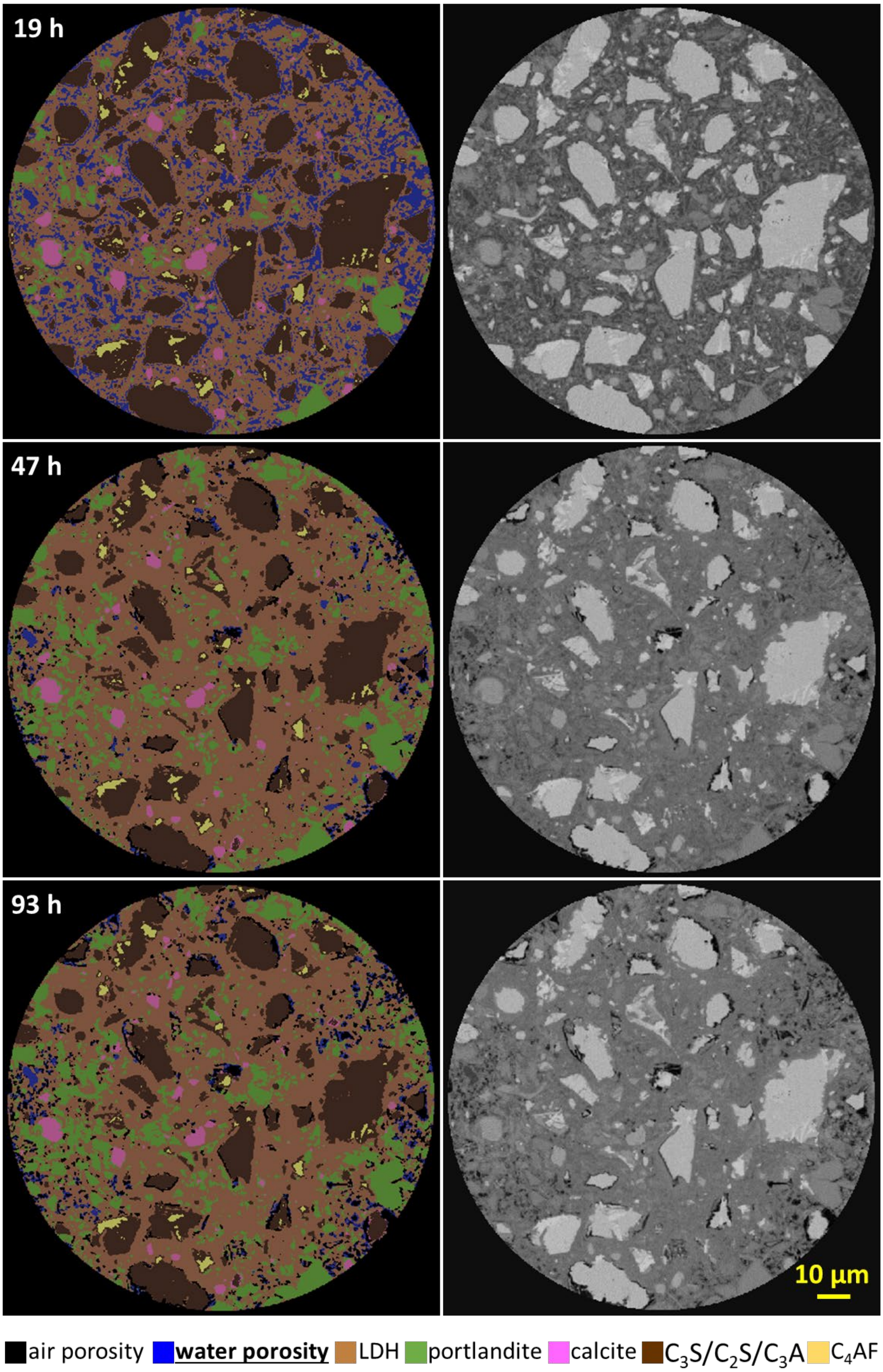

Figure S28. Capillary water porosity evolution, with the obtained spatial resolution (i.e. approximately two voxels). Field of view ~160 µm. (Left) ML segmentation output. (Right) 2D orthoslices of the raw PXCT datasets.

• Supplementary movies

- **Movie-1:** "Summary of 4D nanoimaging of cement hydration" 43 seconds.

  A summarized display of the cement paste hydration evolution as seen by this nanoimaging study. The progress of the different components is displayed after segmentation by Machine-Learning. Moreover, key changes like water porosity evolution or shrinkage development are highlighted on the video by embedded written text.

- **Movie-2:** " C-S-H shell characterization at 19 hours " 17 seconds.

  A video revealing the arrangement of the 3D segmented C-S-H shells through the 19 h nanoimaging dataset.

The size of each short video is standard 640×480 pixels in mp4 format as suggested by Nature journal. Therefore, users can download them quickly.

Article cover image:

Title: "X-ray nanoimaging of a hydrating cement paste at early ages"

Description: The precipitating calcium silicate hydrate shells (blue) surround the dissolving alite particles (yellow) with regions of calcium aluminoferrite highlighted in orange. For better visualisation: only the C-S-H shells in the left part, the three components in the middle region, just the anhydrous cement particles in the right part. The gaps, approximately 500 nm, between the C-S-H shells and the dissolving alite particles are readily visible in the central part of the image.

The cover image is a high resolution 5000×4005 pixels size in .tif format.

Cover image credit: Shiva Shirani, Cover design: Maziar Moussavi

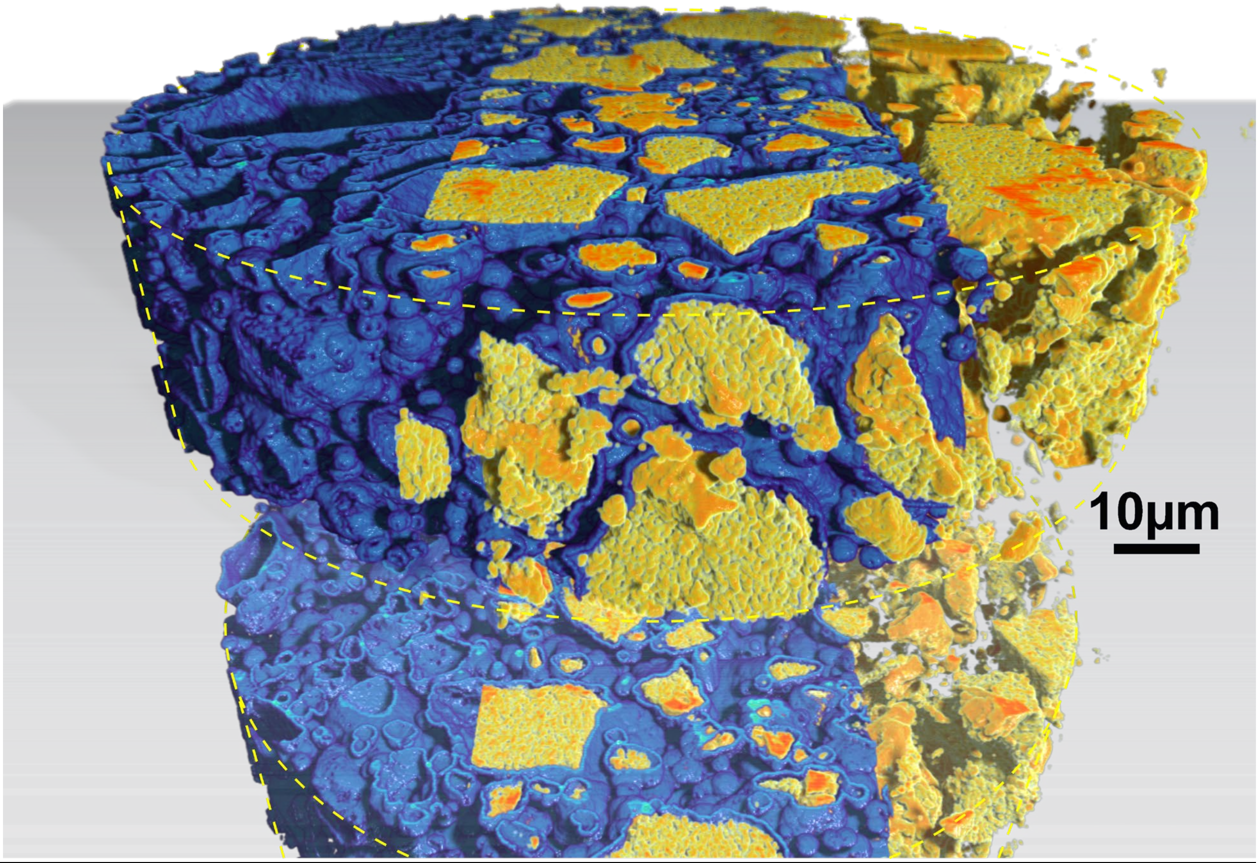

• **Supplementary References**

1. Douissard, P. A. *et al.* A versatile indirect detector design for hard X-ray microimaging. *J. Instrum.* **7**, P09016 (2012).
2. Martin, T. *et al.* LSO-based single crystal film scintillator for synchrotron-based hard X-ray micro-imaging. *IEEE Trans. Nucl. Sci.* **56**, 1412–1418 (2009).
3. Paganin, D., Mayo, S. C., Gureyev, T. E., Miller, P. R. & Wilkins, S. W. Simultaneous phase and amplitude extraction from a single defocused image of a homogeneous object. *J. Microsc.* **206**, 33–40 (2002).
4. Vigano, N., Cloetens, P., di Michiel, M., Rack, A. & Tafforeau, P. Redefining the ESRF tomography software. in *Digital Holography and Three-Dimensional Imaging 2021* paper DF2G.4 (OSA Technical Digest, Optica Publishing Group, 2021). doi:10.1364/DH.2021.DF2G.4.
5. van Heel, M. & Schatz, M. Fourier shell correlation threshold criteria. *J. Struct. Biol.* **151**, 250–262 (2005).
6. Holler, M. *et al.* An instrument for 3D x-ray nano-imaging. *Rev. Sci. Instrum.* **83**, 073703 (2012).
7. Holler, M. *et al.* X-ray ptychographic computed tomography at 16 nm isotropic 3D resolution. *Sci. Rep.* **4**, 3857 (2014).
8. Odstrčil, M., Lebugle, M., Guizar-Sicairos, M., David, C. & Holler, M. Towards optimized illumination for high-resolution ptychography. *Opt. Express* **27**, 14981 (2019).
9. Huang, X. *et al.* Optimization of overlap uniformness for ptychography. *Opt. Express* **22**, 12634 (2014).
10. Dinapoli, R. *et al.* EIGER: Next generation single photon counting detector for X-ray applications. *Nucl. Instruments Methods Phys. Res. Sect. A Accel. Spectrometers, Detect. Assoc. Equip.* **650**, 79–83 (2011).
11. Odstrcil, M., Lebugle, M., Lachat, T., Raabe, J. & Holler, M. Fast positioning for X-ray scanning microscopy by a combined motion of sample and beam-defining optics. *J. Synchrotron Radiat.* **26**, 504–509 (2019).
12. Wakonig, K. *et al.* PtychoShelves, a versatile high-level framework for high-performance analysis of ptychographic data. *J. Appl. Cryst* **53**, 574–586 (2020).
13. Thibault, P., Dierolf, M., Bunk, O., Menzel, A. & Pfeiffer, F. Probe retrieval in ptychographic coherent diffractive imaging. *Ultramicroscopy* **109**, 338–343 (2009).
14. Guizar-Sicairos, M. *et al.* Phase tomography from x-ray coherent diffractive imaging projections. *Opt. Express* **19**, 21345 (2011).
15. Odstrčil, M., Holler, M., Raabe, J. & Guizar-Sicairos, M. Alignment methods for nanotomography with deep subpixel accuracy. *Opt. Express* **27**, 36637–36652 (2019).
16. Diaz, A. *et al.* Quantitative x-ray phase nanotomography. *Phys. Rev. B* **85**, 020104 (2012).
17. Kaestner, A. P. *et al.* Recent developments in neutron imaging with applications for porous media research. *Solid Earth* **7**, 1281–1292 (2016).
18. Donnelly, C. *et al.* Time-resolved imaging of three-dimensional nanoscale magnetization dynamics. *Nat. Nanotechnol.* **15**, 356–360 (2020).
19. Li, X. *et al.* Direct observation of C3S particle dissolution using fast nano X-ray computed tomography. *Cem. Concr. Res.* **166**, 107097 (2023).
20. Cuesta, A. *et al.* Quantitative disentanglement of nanocrystalline phases in cement pastes by synchrotron ptychographic X-ray tomography. *IUCrJ* **6**, 473–491 (2019).
21. Linderoth, O., Wadsö, L. & Jansen, D. Long-term cement hydration studies with isothermal calorimetry. *Cem. Concr. Res.* **141**, 106344 (2021).
22. Trtik, P., Diaz, A., Guizar-Sicairos, M., Menzel, A. & Bunk, O. Density mapping of hardened cement paste using ptychographic X-ray computed tomography. *Cem. Concr. Compos.* **36**, 71–77 (2013).
23. Aranda, M. A. G., Cuesta, A., De la Torre, A. G., Santacruz, I. & León-Reina, L. Diffraction and

crystallography applied to hydrating cements. in *Cementitious Materials: Composition, Properties, Application* (ed. Pöllmann, H.) 31–60 (De Gruyter, 2017). doi:10.1515/9783110473728-003.
24. De la Torre, A. G., Santacruz, I., Cuesta, A., León-Reina, L. & Aranda, M. A. G. Diffraction and crystallography applied to anhydrous cements. in *Cementitious Materials* (ed. Pöllmann, H.) 3–29 (De Gruyter, 2017).
25. Henke, B. L., Gullikson, E. M. & Davis, J. C. X-Ray Interactions: Photoabsorption, Scattering, Transmission, and Reflection at E=50−30,000 eV, Z= 1−92. *At. Data Nucl. Data Tables* **54**, 181–342 (1993).
