# Supplementary material for: 4D nanoimaging of early age cement hydration
Source: Nat Commun. 2023 May 8;14:2652. doi: 10.1038/s41467-023-38380-1 (PMC10167225; doi:10.1038/s41467-023-38380-1)
Supplement: Supplementary file 3 — Description of Additional Supplementary Files [file 41467_2023_38380_MOESM3_ESM.pdf]

Description of Additional Supplementary Files

- File name: Supplementary Movie 1 - Summary of 4D nanoimaging of cement hydration

  Description: A summarized display of the cement paste hydration evolution as seen by this nanoimaging study. The progress of the different components is displayed after segmentation by Machine-Learning. Moreover, key changes like water porosity evolution or shrinkage development are highlighted on the video by embedded written text.

- File name: Supplementary Movie 2 - C-S-H shell characterization at 19 hours

  Description: A movie revealing the arrangement of the 3D segmented C-S-H shells through the 19 h nanoimaging dataset.
